# Marmoset and human trophoblast stem cells differ in signalling requirements and recapitulate divergent modes of trophoblast invasion

**DOI:** 10.1016/j.stem.2024.09.004

**Published:** 2024-09-24

**Authors:** Dylan Siriwardena, Clara Munger, Christopher Penfold, Timo N. Kohler, Antonia Weberling, Madeleine Linneberg-Agerholm, Erin Slatery, Anna L. Ellermann, Sophie Bergmann, Stephen J. Clark, Thomas M. Rawlings, Joshua M. Brickman, Wolf Reik, Jan J. Brosens, Magdalena Zernicka-Goetz, Erika Sasaki, Rüdiger Behr, Florian Hollfelder, Thorsten E. Boroviak

**Affiliations:** 1 Department of Physiology, Development and Neuroscience, University of Cambridge, Cambridge, UK; 2 Centre for Trophoblast Research, University of Cambridge, Cambridge, UK; 3 Wellcome Trust – Medical Research Council Stem Cell Institute, University of Cambridge, Cambridge, UK; 4 Department of Biochemistry, University of Cambridge, Cambridge, UK; 5 Novo Nordisk Foundation Center for Stem Cell Medicine (renew), University of Copenhagen, Copenhagen, Denmark; 6 Altos Labs Cambridge Institute, Cambridge, UK; 7 Epigenetics Programme, Babraham Institute, Cambridge, UK; 8 Wellcome Trust Sanger Institute, Cambridge, UK; 9 Division of Biomedical Sciences, Warwick Medical School, University of Warwick, Coventry, UK; 10 Tommy's National Centre for Miscarriage Research, University Hospitals Coventry and Warwickshire NHS Trust, Coventry, UK; 11 Division of Biology and Biological Engineering, California Institute of Technology, Pasadena, CA, USA; 12 Department of Marmoset Biology and Medicine, Central Institute for Experimental Animals, Kawasaki 210-0821, Japan; 13 German Primate Center, Leibniz-Institute for Primate Research, Göttingen, Germany, and DZHK (German Center for Cardiovascular Research), Göttingen, Germany

## Abstract

Early human trophoblast development has remained elusive due to the inaccessibility of the early conceptus. Non-human primate models recapitulate many features of human development and allow access to early postimplantation stages. Here, we tracked the pre- to postimplantation transition of the trophoblast lineage in superficially implanting marmoset embryos *in vivo*. We differentiated marmoset naive pluripotent stem cells into trophoblast stem cells (TSCs), which exhibited trophoblast-specific transcriptome, methylome, differentiation potential and long-term self-renewal. Notably, human TSC culture conditions failed to support marmoset TSC derivation, instead inducing an extraembryonic mesoderm-like fate in marmoset cells. We show that combined MEK, TGFβ/NODAL and histone deacetylase inhibition stabilizes a periimplantation trophoblast-like identity in marmoset TSCs. By contrast, these conditions differentiated human TSCs towards extravillous trophoblast. Our work presents a paradigm to harness the evolutionary divergence in implantation strategies to elucidate human trophoblast development and invasion.

**Figure:**
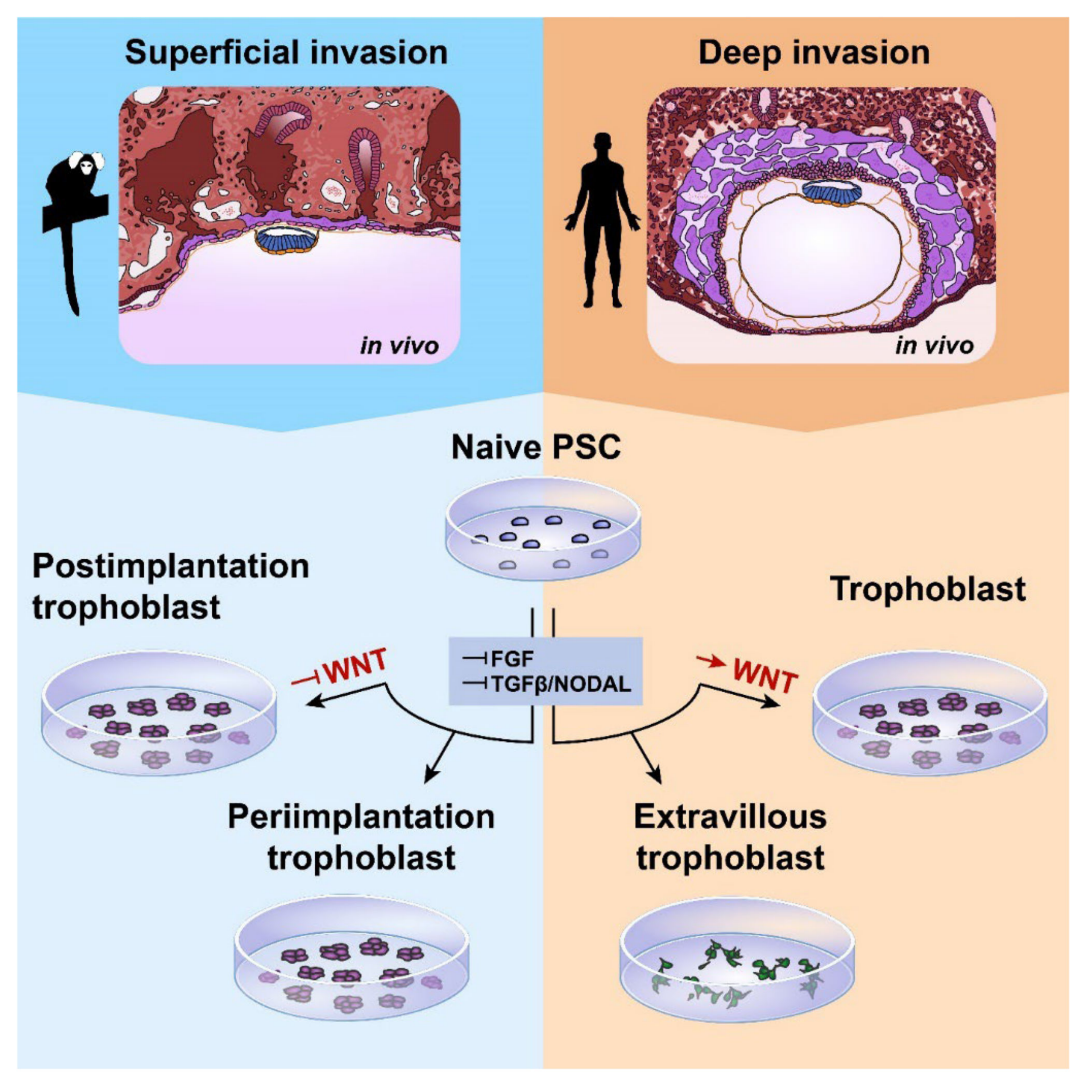

## Introduction

Embryo implantation and placentation are defining hallmarks of eutherian development. The trophoblast lineage mediates these critical tasks and is essential to establish the link between the embryo and the maternal tissues^1^.

All trophoblast lineages originate from the trophectoderm, the outer cell layer of the mammalian preimplantation embryo ^2–5^. At implantation, the trophectoderm undergoes primary syncytialization to form invasive cells that attach and penetrate the luminal epithelium of the uterus^6,7^. In the early stages of human postimplantation development, the trophectoderm differentiates into three major lineages: cytotrophoblast, syncytiotrophoblast and extravillous trophoblast (EVT). Cytotrophoblast is an undifferentiated and proliferative cell population, that, in human, surrounds the entire conceptus and gives rise to multinucleated syncytiotrophoblast and highly migratory EVT cells^4,8^. Primary syncytiotrophoblast are multinucleated cells that arise upon embryo attachment, form lacunae for nutrient exchange and secrete chorionic gonadotropin (CGA, CGB) to sustain the pregnancy ^6,8–15^. EVT cells deeply invade into the uterus, where they play a pivotal role in spiral artery remodelling and immune modulation ^4^.

Abnormalities in trophoblast development and invasion have profound consequences for maternal and fetal health^16^. Insufficient trophoblast invasion can lead to fetal growth restriction and pre-eclampsia^17^, while excessive trophoblast invasion causes placenta accreta spectrum disorders^16,18,19^. Despite the importance of the placenta for healthy pregnancy outcomes, the molecular mechanisms controlling human trophoblast invasion depth and placental development remain ill-understood due to the inaccessibility of early human postimplantation samples.

The derivation of human trophoblast stem cells (TSCs)^20–22^ has provided an essential tool to delineate the mechanisms of cytotrophoblast self-renewal^23^ and differentiation^24–26^. Trophectoderm-like identities can also be induced from naive (preimplantation) pluripotent stem cells (PSCs) by TGFβ/NODAL and FGF/ERK inhibition^25,27–30^. Interestingly, recent studies have demonstrated that human trophoblast culture conditions^20^ promoted not only trophoblast-like identities from naive PSCs, but also extraembryonic mesoderm-like cell types, suggesting some degree of overlap in extraembryonic lineage specification^31^.

Rare *in vivo* datasets from early pregnancy terminations provide essential insights into postimplantation development^32,33^, but trophoblast samples from periimplantation and early gastrulation stages remain elusive. Therefore, the paucity of *in vivo*-developed early postimplantation transcriptional signatures in human has hampered the validation of *in vitro*-generated TSCs from naive PSCs.

Non-human primate models are a promising platform to study early postimplantation primate development. *In vivo* studies in the common marmoset, cynomolgus macaque and rhesus macaque have illuminated the molecular framework governing early postimplantation embryonic development^34–37^. However, trophoblast formation and placentation are subject to evolutionary pressures and therefore vary across mammalian species^7,13,15,38,39^. New World monkeys, Old World monkeys, apes and humans exhibit increasing degrees of trophoblast invasion, respectively. Embryos of the common marmoset (*Callithrix jacchus*), a New World monkey, undergo superficial implantation in the central lumen of the uterus. This implantation mode is more shallow and less invasive compared to interstitial implantation in Great apes (orangutan, gorilla, chimpanzee, human), where the embryo penetrates deeply into the uterus and becomes fully engulfed by the endometrium^4,7,40–42^. We previously proposed that studying embryo implantation in different primate species may provide a model to tackle trophoblast invasion-related disorders^13^. However, TSCs from a New World monkey have remained elusive.

Here, we investigated early marmoset trophoblast development *in vivo* to delineate the impact of implantation and early invasion on trophoblast identity and to establish a molecular framework for the derivation of marmoset TSCs *in vitro*. We determined culture conditions to stabilise periimplantation and early postimplantation trophoblast identities and uncovered a human-specific role for WNT signalling. Marmoset TSCs recapitulated primary syncytium formation and invasive EVT-like differentiation, providing insights into the regulators of trophoblast lineage progression. Our results demonstrate that marmoset TSCs are a powerful resource to capture the evolutionary divergence of primate trophoblast development.

## Results

### Marmoset trophoblast development *in vivo*

**Figure 1:**
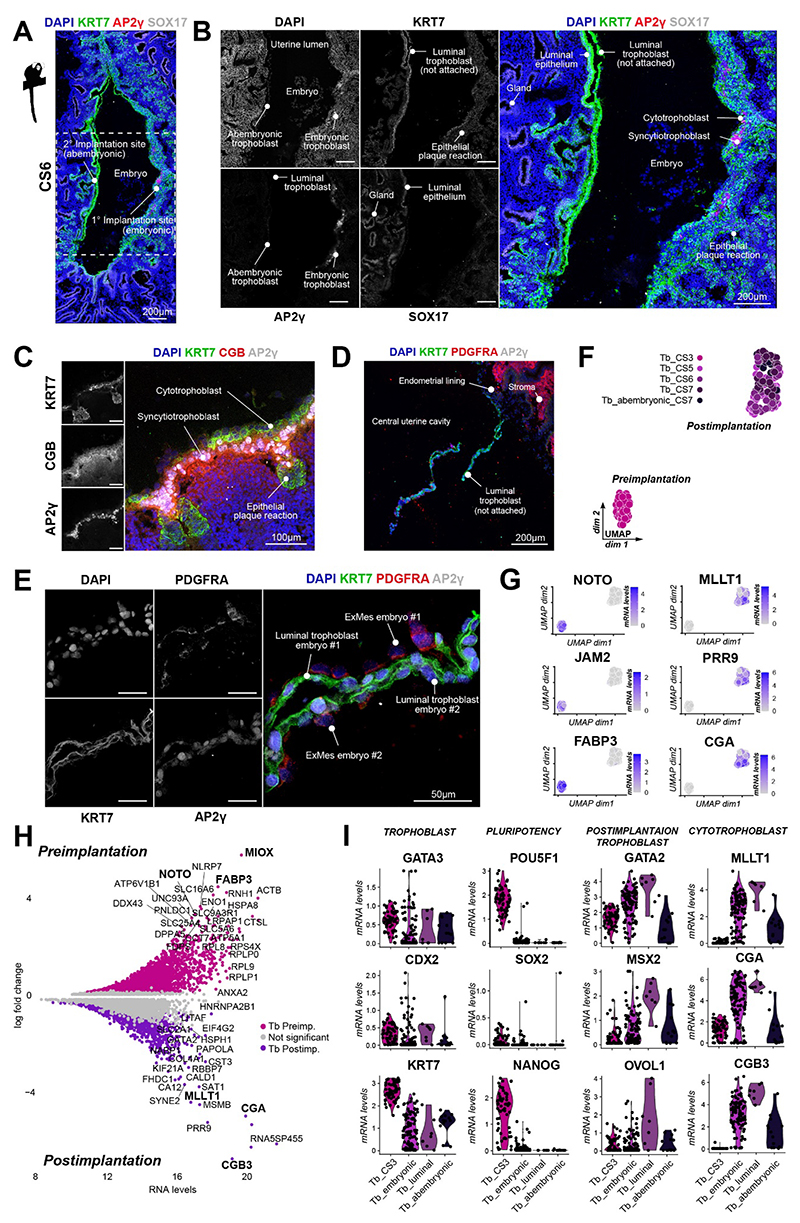
Postimplantation marmoset trophoblast exhibit shallow invasion. (A) Immunofluorescence staining (IF) of CS7 marmoset primary (1°) and secondary (2°) implantation sites. Tile-scanned images were automatically merged by the acquisition software. (B) Expanded view of (A). (C) IF of CS5 implantation site. (D) IF of contact point between luminal trophoblast of twin embryos. (E) Expanded view of (D). (F) UMAP plot of trophoblast lineages. Tb_CS3: trophectoderm; Tb_CS5, Tb_CS6, Tb_CS7: postimplantation embryonic trophoblast; Tb_abembryonic: postimplantation abembryonic trophoblast. (G) UMAP plot from (F) showing normalized log expression. (H) MA plot of differentially expressed genes (DEGs) between trophectoderm (Tb_CS3) and postimplantation trophoblast (Tb_Post). (I) Violin plots of normalized mRNA counts in trophoblast lineages. See also Figure S1.

We recently illuminated early marmoset implantation stages by spatial embryo profiling^34^. To investigate marmoset trophoblast attachment, invasion and early placentation, we performed further analysis of specimens from Carnegie stages (CS) 5-7^34^ with a focus on trophoblast development. At CS5, the polar trophectoderm (proximal to the inner cell mass (ICM)) has adhered to the endometrial lining (primary implantation site) and started to break down the luminal epithelium (Figure S1A,B). We identified a discontinuous KRT7+ cytotrophoblast layer near the primary implantation site, which was interspersed with CGB-secreting multinucleated syncytiotrophoblast (Figure 1A,B,C,S1B). Both, cyto- and syncytiotrophoblast were positive for AP2γ (*TFAP2C*), with syncytiotrophoblast expressing the highest AP2γ levels (Figure 1B,C,S1B). The thickness and density of the AP2γ+ trophoblast layer only marginally increased from CS5 (6-100 µm wide) to CS7 (6-135 µm wide) (Figure 1B,C,S1B,C), demonstrating minimal trophoblast proliferation or invasion immediately after implantation. In contrast, human early postimplantation trophoblast proliferates rapidly, increasing in thickness from 3-90 µm at CS5 to 160-830 µm at CS7 ^43^.

At CS5, the mural trophectoderm (abembryonic trophoblast, distal from the ICM) expanded up to 2 mm to fill the uterine cavity and made loose contact with the opposing uterine wall (Figure S1A,B). By CS6, attached mural trophectoderm breached the luminal epithelium and established a secondary implantation site on the uterine wall opposite to the embryo (Figure 1A,B). AP2γ+ syncytiotrophoblast appeared at the secondary implantation site (14 µm wide) by CS7, but was thinner compared to the primary implantation site (35 µm wide) (Figure S1C,D)^34,39^. In most Old World and New World monkeys, including marmosets, the endometrial epithelium is remodelled to form epithelial plaques at the implantation site^7,34,39,44,45^. This process, which is not observed in Great apes, has been compared to decidualisation in human^10,44^. Interestingly, both primary and secondary embryo implantation induced widespread epithelial plaque reaction, transforming SOX17+ endometrial glands into rounded, densely packed clusters of KRT7+, SOX17- epithelial plaque cells (Figure 1A,B,C).

Outside the primary and secondary implantation site, the mural trophectoderm remained unattached throughout CS5-7. Luminal epithelium adjacent to unattached trophoblast maintained its columnar morphology (Figure 1B). We refer to this unattached postimplantation trophoblast as luminal trophoblast. In one specimen with a twin-pregnancy, we were able to obtain cross-sections at the border between two implanting blastocysts (Figure 1D,E). The luminal trophoblast consisted of AP2γ+/KRT7+ epithelial cells, which were clearly distinguishable from PDGFRα+/AP2γ-/KRT7- extraembryonic mesoderm (ExMes) lining the luminal trophoblast (Figure 1E).

Transcriptome analysis of the pre- to postimplantation transition showed that trophectoderm samples separated from postimplantation trophoblast (Figure 1F). Examination of the postimplantation trophoblast cluster demonstrated no obvious sub-clustering along developmental time (Figure 1F). We identified *NOTO, JAM2* and *FABP3* as enriched in the trophectoderm, while postimplantation trophoblast samples predominantly upregulated *MLLT1, PRR9, CGB3* and *CGA* (Figure 1G,H). Gene ontology (GO)-term analysis of the pre- to postimplantation transition showed differences in WNT signalling and basement membrane organization (Figure S1E).

To identify postimplantation trophoblast lineages, we annotated trophoblast samples according to their location and transcriptomic signature (see Methods). We analysed spatially distinct trophoblast lineages: trophectoderm (preimplantation trophoblast of the blastocyst), embryonic trophoblast (postimplantation, attached trophoblast at the primary implantation site), abembryonic trophoblast (postimplantation, attached trophoblast at the secondary implantation site) and luminal trophoblast (postimplantation, unattached trophoblast) (Figure 1I,S1F-H). Core trophoblast factors *GATA2, GATA3* and *KRT7* were expressed throughout all trophoblast lineages (Figure 1I). The marmoset trophectoderm expressed low levels of pluripotency markers *POU5F1, SOX2* and *NANOG*, which were sharply downregulated in all postimplantation trophoblast samples (Figure 1I). Conversely, syncytiotrophoblast-associated genes such as *CGA* and *CGB3* were upregulated upon implantation (Figure 1H,I)^23^. Human cytotrophoblast-associated transcripts *MSX2* and *OVOL1* were highest in luminal trophoblast (Figure 1I, S1H). Abembryonic trophoblast samples expressed nearly all postimplantation markers, including *MLLT1, NR2F2, CGA* and *CGB3*, albeit at lower levels than embryonic trophoblast (Figure 1I, S1F). This observation is in line with the delay of trophoblast invasion at the secondary implantation site (Figure 1A, B). The interspersed nature of cytotrophoblast and syncytiotrophoblast made it difficult to discriminate between the two lineages. To overcome this challenge, we performed further hierarchical clustering of embryonic trophoblast, which revealed two closely related cell populations (Figure S1I). Differential expression analysis between the two clusters identified them as cytotrophoblast (KRT7+, CDH1+) and syncytiotrophoblast (CGB3+, MLLT1+) (Figure S1J).

We conclude that marmoset trophectoderm expresses residual levels of core pluripotency factors that are extinguished upon implantation. Postimplantation trophoblast gives rise to a discontinuous layer of AP2γ (*TFAP2C*)+/KRT7+ cytotrophoblast and syncytium at the implantation sites, which can be readily demarcated by transcription factors *MLLT1, MSX2* and *OVOL1* as well as pregnancy hormones *CGA* and *CGB3*.

### Marmoset cells differentiate to ExMes in OK conditions

**Figure 2:**
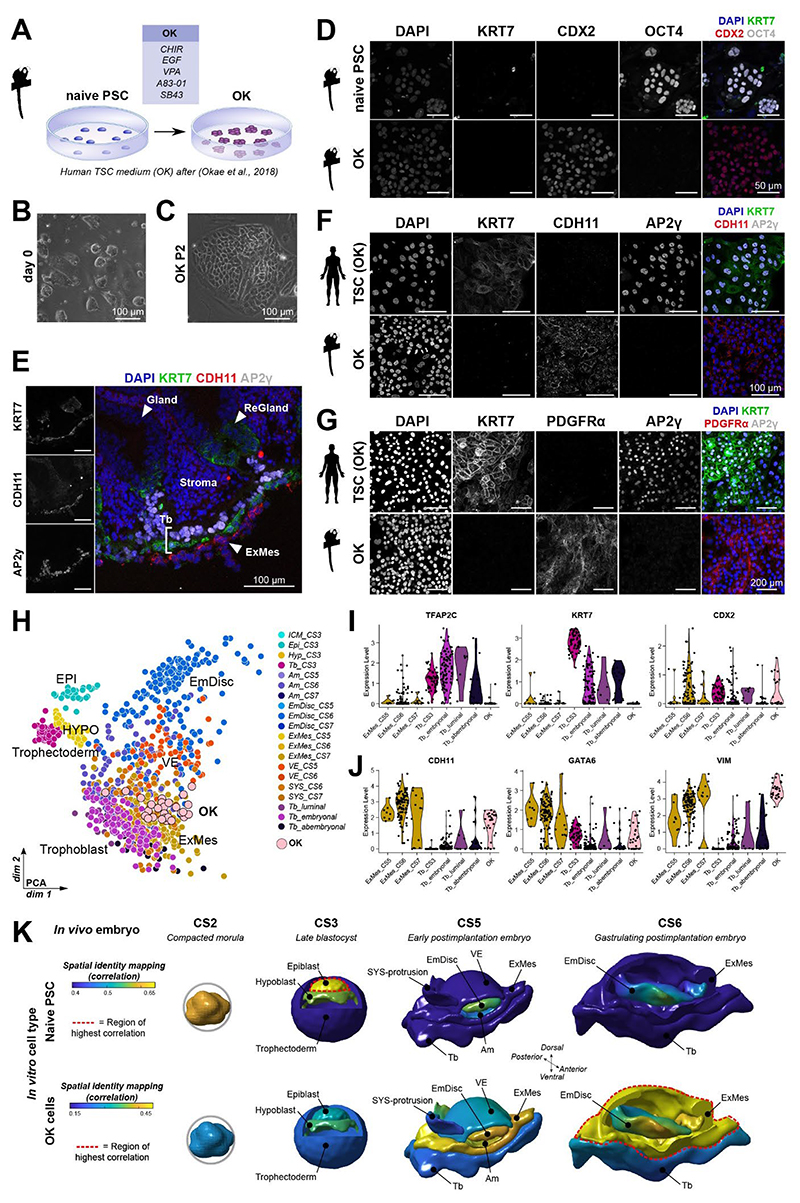
Human TSC culture conditions induce ExMes in marmoset naive PSCs. (A) Schematic of naive marmoset PSC differentiation in OK conditions. (B) Phase contrast image of naive marmoset PSCs. (C) Phase contrast image of passage 2 OK-derived cells. (D) IF of PSCs and passage 2 OK-derived cells. (E-G) IF of (E) *in vivo* CS5 implanting marmoset embryo and (F,G) OK differentiated marmoset and human cells. (H) PCA of *in vivo* marmoset dataset and *in vitro* cells. EPI=preimplantation epiblast, HYPO=hypoblast, EmDisc=embryonic disc, VE=visceral endoderm (I,J) Violin plot of normalized expression of *in vivo* and *in vitro* lineages. (K) Spatial identity mapping of marmoset naive PSCs and OK cells to the embryo. See also Figure S2.

To investigate developmental trajectories of marmoset trophoblast upon implantation, we sought to establish *in vitro* models of peri- and postimplantation trophoblast. Human naive PSCs can be differentiated into extraembryonic lineages including trophoblast, hypoblast and ExMes^25,27,29–31,46,47^. The activation of WNT/EGF and inhibition of TGFβ/HDAC/ROCK in human TSC (OK) medium^20^ promotes a trophoblast-like state in naive human PSCs^25,47^. We recently reported the establishment of preimplantation epiblast-like marmoset naive PSCs^34^. To examine whether human trophoblast conditions can generate marmoset TSCs from naive PSCs, we switched culture conditions to human trophoblast (OK) medium^20^ (Figure 2A). The majority of dome-shaped naive PSC colonies flattened into epithelial colonies with varying morphologies at day 5 (Figure 2B,S2A). By passage 2, the dome-shaped colonies disappeared, resulting in a homogenous culture of flat epithelial colonies (marmoset OK cells) that expressed trophoblast markers, including CDX2 and *TFAP2C* and downregulated the core pluripotency factor OCT4 (Figure 2C,D,S2B). However, marmoset OK cells lacked expression of core trophoblast markers, including *GATA2, GATA3* and *CGA*. Moreover, we observed upregulation of hypoblast and ExMes lineage markers *GATA6* and *PDGFRα* (Figure S2B). Immunofluorescence of marmoset embryos *in vivo* demonstrated exclusive expression of PDGFRα and CDH11 within the ExMes (Figure 1E,2E)^34^. Marmoset OK cells expressed both PDGFRα and CDH11 and lacked expression of KRT7, in contrast to human OK TSCs^20^ (Figure 2F,G). These findings suggest that marmoset OK cells exhibit features of ExMes.

To further interrogate the developmental identity of marmoset OK cells, we performed full-length single-cell transcriptome profiling and integrated OK cells into our updated marmoset embryo datasets (Figure 1)^34,48^. Global dimensionality reduction methods revealed that OK cells clustered closest with postimplantation ExMes cells (Figure 2H,S2C). Global correlation analysis showed that marmoset OK cells had the highest similarity with ExMes at CS6 and expressed known human ExMes markers (Figure S2D,E)^31^. Consistent with RT-qPCR data, OK cells expressed low levels of trophoblast markers *TFAP2C* and *KRT7* in comparison to pre- and postimplantation trophoblast lineages *in vivo* (Figure 2I). Notably, *CDX2* is highly expressed in both trophectoderm and ExMes. OK cells upregulated *CDX2* and various other ExMes markers, including *CDH11, GATA6* and *VIM* (Figure 2I,J). To establish the regional correlation of OK single-cell transcriptomes to the embryo, we performed spatial-identity-mapping to the embryo (Figure 2K). Naive PSCs correlated to the preimplantation epiblast, as previously reported^34^. In contrast, OK cells showed highest spatial correlation scores with postimplantation ExMes at CS5 and CS6 (Figure 2K). These results are consistent with the recent finding that human naive PSCs can generate both trophoblast and extraembryonic mesoderm in OK conditions^31^. Interestingly, a small number of OK cells clustered closely with postimplantation trophoblast (Figure 2H). To determine if OK conditions promoted a trophoblast phenotype during early differentiation of naive marmoset cells, we monitored KRT7 expression during the first 18 days of OK differentiation (Figure S2F,G). We observed that small KRT7+ subpopulations arose by day 12, which were lost in mature OK cultures (day 50+) (Figure S2F,G). Thus, human OK TSC medium does not allow direct TSC derivation from naive PSCs in marmoset and promotes ExMes lineage entry instead.

### Combined WNT-, NODAL- and FGF/ERK-inhibition is required for marmoset TSCs

**Figure 3:**
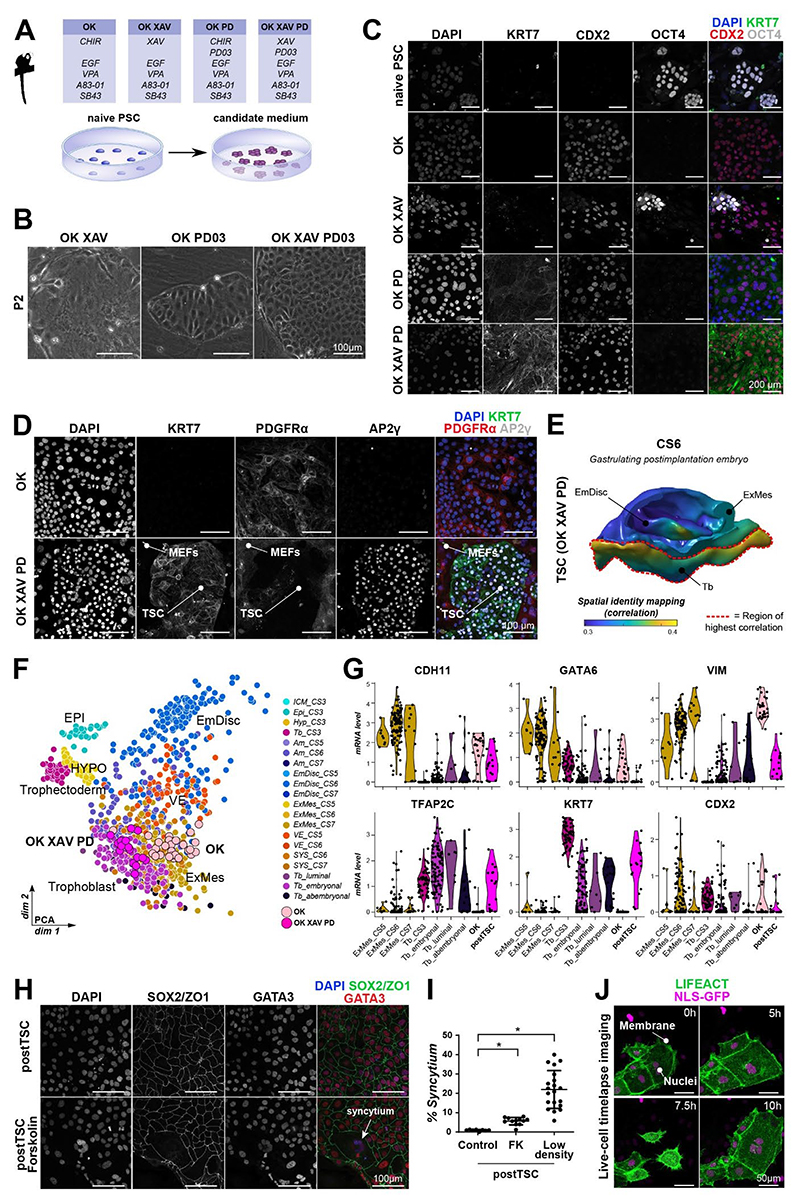
Additional WNT and FGF/ERK inhibition stabilises postimplantation trophoblast identity in marmoset. (A) Schematic of naive marmoset PSC differentiation in variations of OK conditions. (B) Phase contrast images of naive PSCs and differentiated cells at passage 2 in indicated conditions. (C) IF of marmoset cells in indicated conditions. (D) IF of passage 2 OK and OK XAV PD-derived cells cultured on MEFs. MEFs constitutively express PDGFRα. (E) Spatial identity mapping of marmoset postTSCs to the embryo. (F) PCA of *in vivo* marmoset dataset and *in vitro* cells. EPI=preimplantation epiblast, HYPO=hypoblast, EmDisc=embryonic disc, VE=visceral endoderm. (G) Violin plot of normalized expression in *in vivo* and *in vitro* lineagess. (H) IF of postTSC differentiation with forskolin. (I) Quantification of multinucleated cell frequency. Each point represents a single frame where the proportion of multinucleated to single nucleated cells were counted. Significance was calculated using a two-tailed Mann–Whitney test. N=3. Error bars represent mean + SD (*: p ≤0.05). FK: Forskolin (J) Live imaging of tagged marmoset TSCswith nuclear GFP (red) and F-actin binding LifeACT (green). Images were taken every 30 minutes. See also Figure S3.

Human OK TSC medium stimulates WNT and EGF-signalling and inhibits TGFβ/NODAL-signalling ^20,22^. Moreover, trophectoderm fate can be readily induced in human naive PSCs by inhibition of FGF/ERK and TGFβ/NODAL signalling in the absence of WNT stimulation ^27,29^. In the embryo, we observed dynamic changes of WNT-signalling in the pre- to postimplantation transition of marmoset trophoblast development (Figure S1D). To identify the signalling requirements for trophoblast identity, we modulated WNT and FGF/ERK in OK medium during marmoset TSC derivation from naive PSCs on inactivated mouse embryonic fibroblast (MEFs) (Figure 3A). OK with the WNT inhibitor XAV939 (OK XAV), OK with the FGF/ERK inhibitor PD0325901 (OK PD) and OK with WNT and FGF/ERK inhibition (OK XAV PD) each induced flattening and epithelialization of naive PSCs after 5 days (Figure 3B). Immunocytochemistry revealed that OK XAV retained a proportion of OCT4+ cells and lacked KRT7 expression (Figure 3C,S3A,B). OK PD efficiently downregulated OCT4 and mildly upregulated KRT7 (Figure 3C,S3A,B), suggesting that MEK inhibition partially suppresses ExMes differentiation. OK XAV PD exhibited high levels of KRT7, expressed CDX2 and was negative for OCT4 (Figure 3C,S3A,B). Considering that KRT7 was strongly expressed in trophoblast *in vivo*, we decided to further investigate OK XAV PD cells.

Marmoset OK XAV PD cells could be stably maintained for at least 14 passages and were readily established in a second, independent marmoset PSC line (Figure S3C). In contrast to OK cells, OK XAV PD cells did not express the ExMes marker PDGFRα and upregulated the trophoblast marker AP2γ (*TFAP2C*) (Figure 3D). Hence, OK XAV PD cells recapitulated the hallmarks of trophoblast stem cells, exhibiting long-term self-renewing capability and lineage markers expression specific to human TSCs and marmoset cytotrophoblast.

To assess the developmental authenticity of OK XAV PD cells, we performed single-cell transcriptome profiling. Spatial-identity-mapping and integrated analysis showed that OK XAV PD cells did not correspond to ExMes but were most similar to postimplantation trophoblast in the marmoset embryo (Figure 3E, 3F,S3D). Therefore, we denoted OK XAV PD cells as postimplantation trophoblast stem cells (postTSCs). Notably, ExMes-associated genes, including *CDH11, GATA6, VIM* were extinguished in postTSCs, while trophoblast factors *KRT7* and *TFAP2C* were expressed at the same levels as in the postimplantation trophoblast of the marmoset conceptus (Figure 3G). *CDX2* levels were reduced compared to OK cells, ExMes and preimplantation trophoblast (trophectoderm), but similar to postimplantation trophoblast. Correlation analysis showed that marmoset postTSCs corresponded to postimplantation trophoblast at CS6 (Figure S3E), similar to human TSCs ^23,49^.

A defining hallmark of trophoblast cells is their capacity to form syncytiotrophoblast^3,38,50^. Human TSCs are capable of differentiation into a multinucleated syncytium upon cAMP stimulation with forskolin^20^. Equally, marmoset postTSCs formed multinucleated cysts in the presence of forskolin (Figure 3H,I). Low cellular densities also promoted syncytium formation (Figure 3I, S3F). Moreover, marmoset syncytiotrophoblast genes were enriched in a subset of sequenced postTSCs, suggesting a propensity for syncytiotrophoblast differentiation in postTSCs (Figure S3G). To determine the mechanism by which multinucleated syncytium is formed *in vitro*, we tracked syncytium formation by live-cell imaging. We generated nuclear GFP and F-actin mCherry-labelled PSCs (NLS-GFP LIFEACT) and derived marmoset postTSCs. Time lapse imaging showed that marmoset postTSCs predominantly formed syncytium via endoreduplication (Figure 3J, S3H, Supp.Video1), where mitosis occurred without cytokinesis. On rare occasions, cells would surround the nucleus of another cell, suggesting that cellular fusion may also occur during marmoset syncytium formation (Figure S3I).

We conclude that marmoset postTSCs closely resemble early postimplantation trophoblast *in vivo*, are capable of long-term self-renewal and give rise to syncytiotrophoblast.

### PAVS stabilises a periimplantation trophectoderm-like state

**Figure 4:**
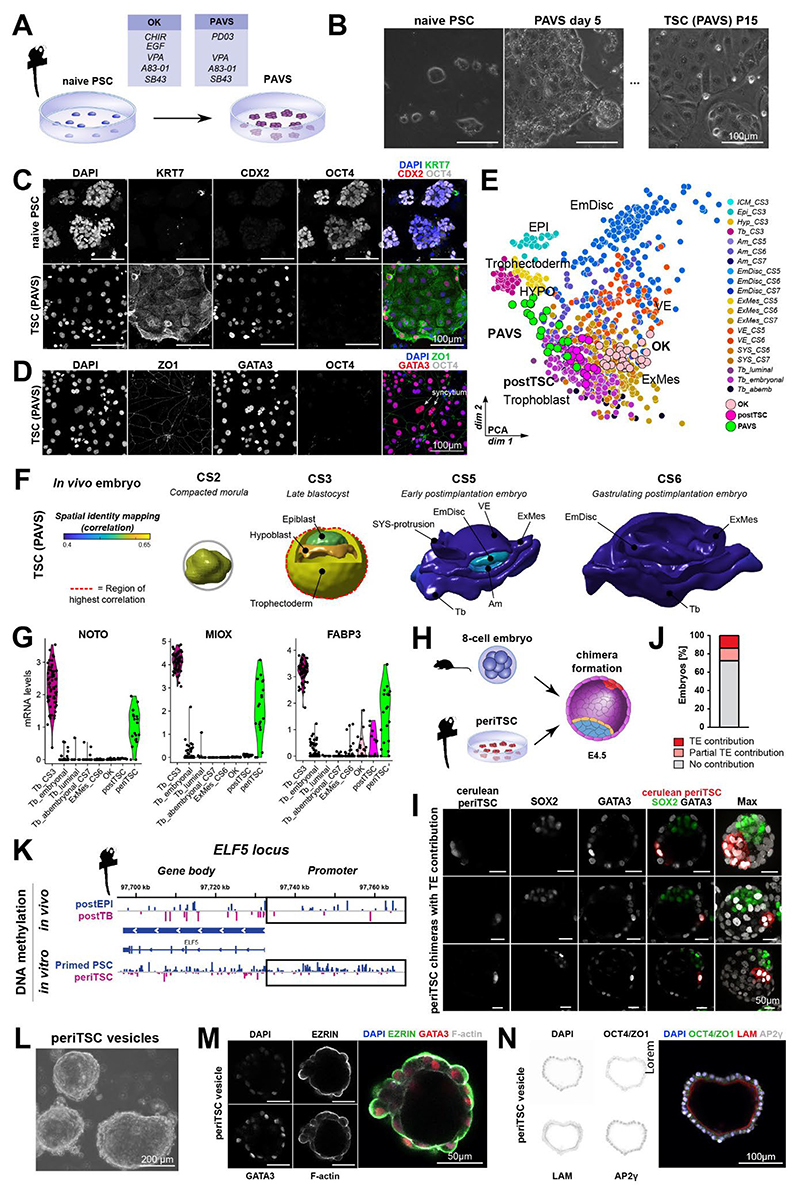
PAVS promotes a periimplantation trophoblast phenotype. (A) Schematic of naive marmoset PSC differentiation in PAVS. (B) Phase contrast images of naive PSCs and differentiated cells at day 5 and passage 15 in PAVS. (C) IF of marmoset cells in indicated conditions. (D) IF of multinucleated cells in PAVS. (E) PCA of *in vivo* marmoset dataset and *in vitro* cells. EPI=preimplantation epiblast, HYPO=hypoblast, EmDisc=embryonic disc, VE=visceral endoderm. (F) Spatial identity mapping of marmoset periTSCs to the embryo. (G) Violin plot of normalized expression in *in vivo* and *in vitro* lineages. (H) Schematic of cross-species chimeras with mouse embryos and marmoset periTSCs. (I) IF of E4.5 chimeric mouse embryos aggregated with tagged periTSCs. (J) Quantification of lineage contribution of periTSCs in chimeric mouse embryos (n=44). Embryos were collected from 7 different mice. (K) Relative methylation of the ELF5 locus in periTSCs and primed PSCs in comparison to *in vivo* postimplantation trophoblast and embryonic disc. (L) Phase contrast image of periTSCs spontaneously forming spheroids in 2D culture. (M,N) IF of periTSC-spheroids. F-actin stain: phalloidin.. LAM: laminin. See also Figure S4.

Human naive PSCs are capable of differentiation into trophectoderm-like cells upon FGF/ERK and TGFβ/NODAL inhibition ^27,29^. To test whether a developmentally earlier, preimplantation trophectoderm-like state could be stabilised, we cultured marmoset naive PSCs in media containing the MEK inhibitor PD0325901, two TGFβ/NODAL inhibitors (A83-01 and SB431542) and valproic acid (VPA) (Figure 4A). Naive PSCs in PAVS medium differentiated into flat, epithelial colonies, which could be sustained in culture for more than 15 passages on MEFs (Figure 4B). Immunofluorescence showed that cells cultured in PAVS medium expressed high levels of trophoblast markers KRT7, CDX2 and GATA3, in the absence of OCT4 (Figure 4C,D). Consistent with this trophoblast lineage marker profile, PAVS cultures occasionally formed multinucleated syncytium that expressed the syncytiotrophoblast marker CGB (Figure S4A). We confirmed trophoblast induction in PAVS medium in a second independent marmoset PSC line.

Microvilli are characteristic for marmoset preimplantation trophoblast and thought to play an important role in primate embryo implantation ^6,7^. Scanning electron microscopy of PAVS cells revealed extensive microvilli formation, while naive PSCs did not form microvilli (Figure S4B). Examination of cell colony edges showed that microvilli were confined to the apical surface, as observed in implanting primate embryos (Figure S4B) ^6,7^

To assess the developmental identity of PAVS-derived TSCs at the transcriptome level, we performed single-cell profiling and mapped PAVS cells to the spatial transcriptomic embryo dataset. PCA placed PAVS cells in between trophectoderm and early postimplantation trophoblast samples (Figure 4E). Spatial-identity-mapping revealed greatest correlation scores to trophectoderm and lower correlation to postimplantation trophoblast (Figure 4F). UMAP showed similar results, with some PAVS cells clustering with trophectoderm at CS3 (Figure S4C). Global correlation analysis placed PAVS cells with trophectoderm, in contrast to postTSCs, which more closely correlated with postimplantation trophoblast (Figure S4D). Therefore, we annotated PAVS TSCs as periimplantation trophoblast-like TSCs (periTSCs). periTSCs lacked expression of ExMes-associated transcripts and showed robust trophoblast gene expression (Figure S4E). Importantly, periTSCs upregulated preimplantation-specific mRNAs, including *NOTO, MIOX* and *FABP3*, in contrast to postTSCs and OK cells (Figure 4G). To examine whether periTSCs are able to contribute to trophectoderm, we generated cross-species aggregation chimeras with mouse embryos and marmoset periTSCs (Figure 4H). periTSCs contributed exclusively to the trophectoderm with an overall efficiency of 27% (13.5% and 13.5% for complete and partial trophectoderm contribution, respectively) (Figure 4I,J,S4F,G).

Human TSCs exhibit lower global DNA-methylation levels compared to primed PSCs and somatic lineages^20^. Little is known about the methylation dynamics in marmoset pre- and postimplantation development. Consequently, we generated single-cell bisulfite sequencing samples of marmoset *in vivo* postimplantation trophoblast and embryonic disc. Furthermore, we performed bisulfite sequencing on bulk populations of *in vitro* marmoset naive PSCs, primed PSCs and periTSCs. Global CpG methylation levels were lowest in naive PSCs and highest in primed PSCs (Figure S4H), consistent with reported differences between naive and primed pluripotent states in human^51,52^. periTSCs exhibited slightly lower DNA methylation levels compared to primed PSCs and characteristic hypomethylation of the *ELF5* promoter region, consistent with the dynamics observed *in vivo* between the postimplantation trophoblast and the embryonic disc (Figure 4K,S4H,I)^53^. Interestingly, *ELF5* hypomethylation was also observed in naive PSCs, which suggests that some trophoblast factors remain unmethylated in the preimplantation epiblast (Figure S4J). *In vivo* trophoblast and periTSCs exhibited similar hypomethylation patterns for characteristic trophoblast genes such as *KRT7* (Figure S4K). To investigate trophoblast-specific methylation features, we determined differentially methylated genes in naive PSCs versus periTSCs. periTSCs exhibited higher methylation levels of pluripotency factors *POU5F1, NANOG* and *KLF17* (Figure S4I). *SMAD2* and the amnion-specific gene *VTCN1* were also more highly methylated in periTSCs (Figure S4I) ^54–57^, suggesting trophoblast-specific DNA methylation of epiblast- and amnion-related transcripts.

Human and non-human primate PSCs harbour the potential to differentiate into amnion^34,55,56,58–60^. Marmoset amnion *in vivo* exhibits a flat squamous epithelial morphology and expresses several trophoblast-associated genes, including *TFAP2C* and *GATA2*^34^. To determine whether periTSCs or postTSCs expressed molecular features of amnion, we extracted a comprehensive panel of markers that discriminate between amnion and trophoblast *in vivo* (Figure S4M). Both periTSCs and postTSCs expressed the majority of trophoblast-associated genes and lacked amnion-related transcripts, including *VTCN1* and *POU5F1* (Figure S4L), consistent with previous global dimensionality reduction methods (Figure 3F,4E,S3D,S4C). Immunofluorescence confirmed that periTSCs do not express VTCN1 at a protein level (Figure S4M). The addition of BMP4 induced VTCN1 expression (Figure S4M), in line with the reported role of BMP signalling for amnion formation^34,55,56,58,59^.

We observed that periTSCs, when passaged or cultured at higher densities, formed free-floating vesicles that could be maintained in hanging drops for at least five days (Figure 4L,S4O). Immunocytochemistry of periTSC vesicles showed robust expression of the trophectoderm marker GATA3 (Figure 4M). EZRIN and F-actin localized to the outer membrane of periTSC vesicles, which demonstrated that periTSC derived trophoblast spheroids (Tb-spheroids) exhibit an outward facing apical domain, similar to the trophectoderm of the blastocyst (Figure 4M). Consistent with this result, Laminin was found on the inner membrane of the POU5F1-/AP2γ+/ZO1+Tb-spheroids (Figure 4N).

We conclude that PAVS induces a periimplantation trophectoderm-like TSC state, as determined by transcriptome and methylome profiling, chimeric contribution to the trophectoderm of mouse blastocysts and spontaneous syncytium and trophectoderm-like spheroid formation.

### *CDX2* overrides naive and primed self-renewing culture conditions

**Figure 5:**
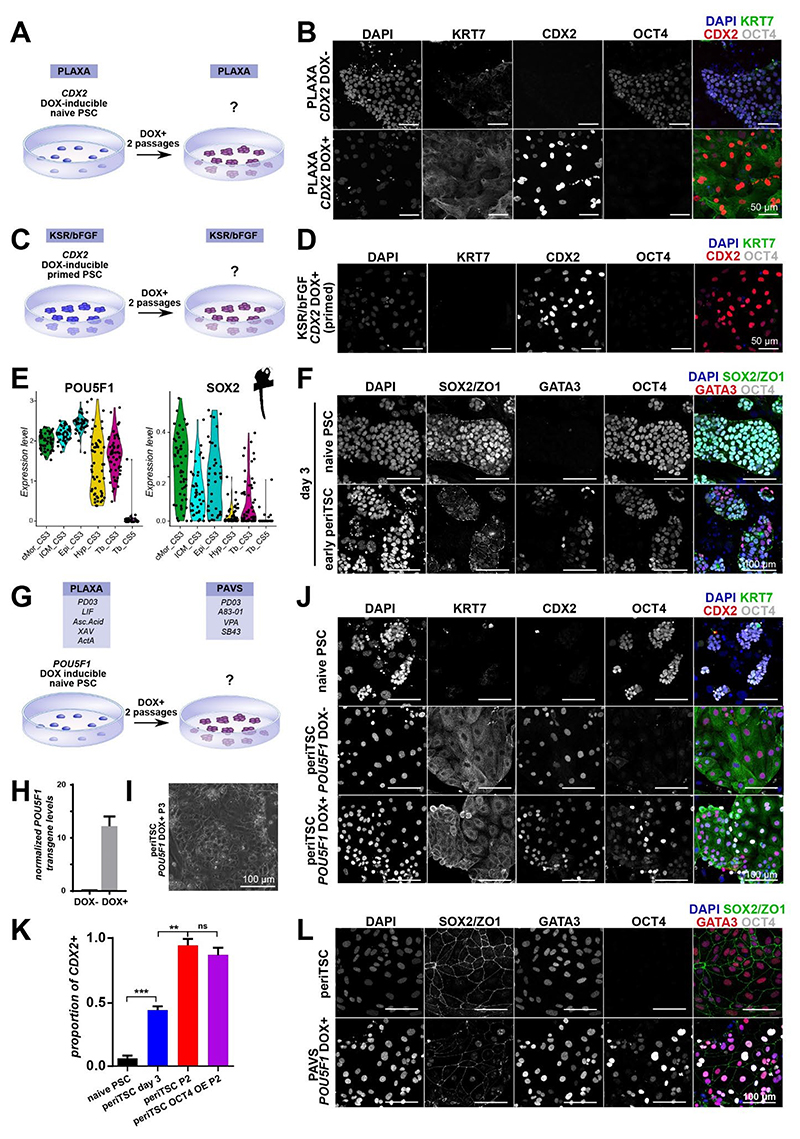
OCT4 does not inhibit trophoblast formation in the marmoset. (A) Schematic of CDX2 overexpression in naive marmoset PSCs for 2 passages. (B) IF of CDX2 overexpression in naive PSCs. (C) Schematic of CDX2 overexpression in primed marmoset PSCs for 2 passages. (D) IF of CDX2 overexpression in primed PSCs. (E) Violin plot of normalized expression in preimplantation marmoset lineages. (F) IF of PAVS-differentiated cells at day 3. (G) Schematic of OCT4 overexpression in naive PSCs cultured in naïve medium (PLAXA). Overexpression was maintained for 2 passages unless stated otherwise. (H) qPCR of overexpression-specific OCT4 expression. (I) Phase contrast image of PAVS-differentiated cells from OCT4 overexpressing naive PSCs at passage 3. Overexpression was maintained for 3 passages. (J) IF of OCT4 overexpression in PSCs and periTSCs in indicated conditions. (K) Quantification of CDX2+ cells in (J). Threshold was determined by 2 times the standard deviation of CDX2 expression in PSCs. Significance was calculated using a Kruskal–Wallis followed by Dunn's multiple comparison test. N=2. (* - p<0.05). (L) IF PAVS-derived cells from OCT4 overexpressing naive PSCs at passage 2. See also Figure S5.

In mouse, CDX2 induces trophoblast lineage entry via the repression of pluripotency factors^61–63^. In particular, CDX2-mediated downregulation of *Pou5f1* (OCT4) is a key juncture during mouse trophectoderm specification^64^. To examine the effect of the trophectoderm transcriptional program on pluripotency in the marmoset, we generated doxycycline-inducible *CDX2* overexpression PSCs lines by PiggyBac transposition (Figure 5A,S5A,B). *CDX2* overexpression for 2 passages in naive PSCs promoted trophoblast identity, even in self-renewing naive PSCs culture conditions (Figure 5A,B). *CDX2*-overexpressing PSCs in naive culture conditions exhibited similar morphology to periTSCs and were maintained beyond passage 3 with CDX2 overexpression (Figure 5A,B). Notably, OCT4 was downregulated in *CDX2* overexpressing cells in naive PSCs culture conditions (Figure S5C). This suggests that CDX2 induction extinguishes OCT4 expression in marmoset PSCs.

To test if CDX2 can promote a trophoblast-like phenotype in primed (postimplantation-like) pluripotency, we assessed *CDX2* overexpression in self-renewing primed PSC culture conditions (KSR/bFGF) (Figure 5C, S5D). CDX2 overexpression in primed cells led to a decrease in OCT4 expression and differentiation (Figure 5D). However, DOX+ cells remained KRT7-, suggesting differentiation into other lineages (Figure 5D). A subset of *CDX2* overexpressing PSCs in KSR/bFGF expressed TBXT+, which may indicate acquisition of a primitive streak-like mesodermal cell identity (Figure S5E)^34,36,65^. We conclude that CDX2 overrules pluripotency maintenance conditions and promotes trophoblast-like identity in naïve, but not primed, marmoset PSCs.

### *POU5F1* does not restrict marmoset trophoblast differentiation *in vitro*

Analysis of pre- and postimplantation-specific trophoblast signatures (Figure 1) identified the presence of pluripotency factors in marmoset trophectoderm *in vivo*, including *POU5F1* (Figure 5E,S5F). This is in contrast to mouse, where *Pou5f1* (OCT4) is rapidly downregulated in the trophectoderm^66^. Therefore, we set out to examine the role of *POU5F1* in marmoset trophoblast differentiation. To elucidate *POU5F1* dynamics during TSC derivation, we performed lineage marker analysis at day 3 during periTSCs differentiation from naive PSCs. SOX2 was downregulated in the majority of cells, however OCT4 was present in all cells, including early GATA3+ and KRT7+ trophoblast cells (Figure 5F,S5H-J). Image quantification showed no anti-correlation (R = 0.1066) between OCT4 and GATA3 expression (Figure S5J). Equally, CDX2 started to become upregulated in a subset of OCT4+ cells (Figure S5G). To test whether *POU5F1* upregulation and sustained expression would prevent differentiation into trophoblast-like cells, we generated marmoset doxycycline-inducible *POU5F1* overexpression PSCs (Figure 5G). We confirmed tight control over the *POU5F1*-transgene (Figure 5H) and derived periTSCs in the presence and absence of doxycycline (DOX+/-) (Figure 5I,J). In the absence of DOX (DOX-), *POU5F1*-inducible naive PSCs converted into KRT7+/CDX2+/POU5F1- periTSCs (Figure 5J), similar to wildtype cells (Figure 4C). In the presence of DOX (DOX+) in PAVS for 2 passages, *POU5F1*-inducible naive PSCs equally flattened out, acquired a trophoblast-like morphology and could be readily propagated (Figure 5I). Sustained *POU5F1* expression did not interfere with the robust expression of trophoblast markers KRT7, GATA3 and CDX2 (Figure 5J-L). We conclude that *POU5F1* does not inhibit trophoblast-specific gene expression *in vitro*.

### Marmoset trophoblast stem cells recapitulate features of superficial attachment

**Figure 6:**
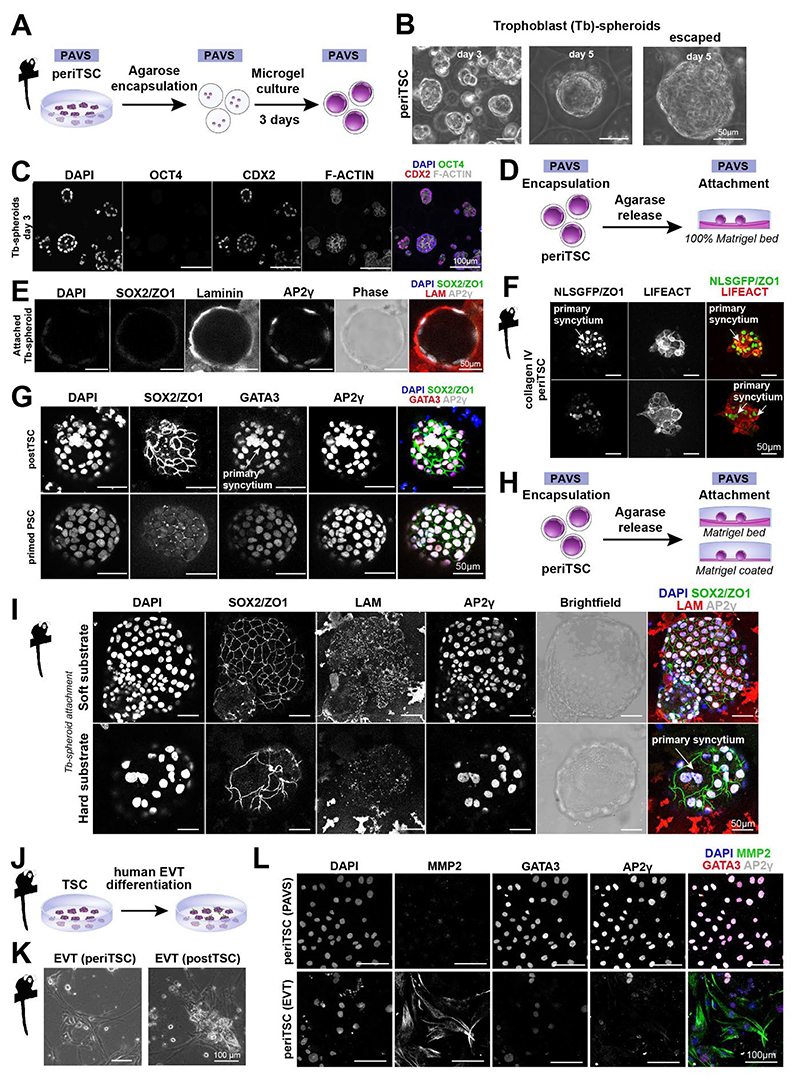
Marmoset TSCs recapitulate characteristics of superficial implantation and early invasion. (A) Schematic of Tb-spheroid formation by encapsulation. (B) Phase contrast images of encapsulated Tb-spheroids at day 3 and 5. escaped: structures that have broken out of agarose microgels and are growing in suspension. (C) IF of marmoset Tb-spheroids at day 3. F-actin stain: phalloidin. (D) Schematic of Tb-spheroid invasion assay. (E) IF of attached marmoset Tb-spheroids on Matrigel beds. (F) IF of NLS-GFP and LifeACT (actin stain) overexpressing Tb-spheroids on a collagen-coated surface. (G) IF of attaching primed marmoset PSC and postTSC spheroids on a Matrigel-coated surface. (H) Schematic of Tb-spheroids released onto ECM of varying thickness. (I) IF of marmoset Tb-spheroids attaching on gels of varying thickness. (J) Schematic of TSC differentiation in human EVT conditions. LAM: laminin. (K) Phase contrast images of periTSCs (PAVS) and postTSCs (OK XAV PD) differentiated in human EVT conditions for 6 days. (L) IF of periTSCs (PAVS) before and after human EVT differentiation. See also Figure S6.

A central function of trophectoderm is to form a vesicle that is capable of implantation. We previously established a microfluidic workflow to encapsulate PSCs into monodisperse agarose microgels^56,67,68^. To set up a platform for trophoblast invasion of Tb-spheroids, we encapsulated either marmoset periTSCs or human OK TSCs in agarose microgels (Figure 6A,B,S6A). Both marmoset periTSCs and human OK TSCs^20^ formed Tb-spheroids in agarose gels from day 3 onwards that continued to expand, resulting in many structures escaping from the microgels by day 5 (Figure 6B). Marmoset and human Tb-spheroids robustly expressed trophoblast markers CDX2 and AP2γ and lacked the pluripotency factor SOX2 (Figure 6C,S6B).

To assess marmoset Tb-spheroid implantation *in vitro*, we allowed spheroids to attach on a Matrigel bed after release from the microgels (Figure 6D). Marmoset Tb-spheroids maintained regular spherical shapes and expanded lumina (Figure 6E,S6D,S6E), consistent with the superficial implantation mode of marmoset embryos (Figure 1A) where the blastocyst expands to fill the uterine cavity. In contrast, human Tb-spheroids exhibited an irregular and deflated morphology (Figure S6C-E). These findings suggest that marmoset TSCs recapitulate some morphological aspects of superficial attachment (Figure S6F).

Primate trophectoderm differentiates into primary syncytium upon contact with the uterine epithelium *in vivo*^69^. To evaluate the ability of Tb-spheroids to recapitulate primary syncytium formation upon attachment, Tb-spheroids were transferred to adherent culture conditions. Tb-spheroids formed a multinucleated syncytium upon contact with thinly coated Matrigel or collagen IV (Figure 6F,G). We confirmed that syncytium formation was specific to TSCs by plating marmoset primed PSC-derived spheroids, which did not give rise to multinucleated cells (Figure 6G). Notably, attachment of Tb-spheroids on thicker beds of Matrigel or collagen IV did not form syncytium (Figure 6H,I,S6G), suggesting that substrate stiffness may play a role in the regulation of primary syncytium formation.

Migratory EVT differentiation is essential for immune, endometrial and vessel remodelling during placentation. Despite significantly delayed trophoblast invasion in the marmoset compared to human, migratory trophoblast cells have been observed in the marmoset endometrium and uteroplacental arteries *in vivo*^42^. To examine if marmoset TSCs are able to differentiate into invasive trophoblast, we cultured periTSCs and postTSCs in human EVT conditions^20^ (Figure 6J). Supplementation of NRG1 and ECM together with TGFβ/NODAL inhibition robustly induced epithelial-mesenchymal transition and expression of the EVT marker MMP2 by day 6, similar to human EVT differentiation (Figure 6K,L, S6I-K). The human EVT marker HLA-G showed very limited conservation in the marmoset (Figure S6H)^70,71^. To determine the migratory potential of marmoset EVT-like cells, we performed transwell migration assays. Marmoset EVT differentiation increased migratory behaviour, similar to EVT differentiation in human (Figure S6L). This result suggests at least partial conservation of EVT specification in human and marmoset.

Together, these experiments show that marmoset periTSCs undergo superficial attachment, form primary syncytia and are capable of EVT differentiation, thus recapitulating important aspects of New World monkey trophoblast development.

### WNT stimulation is required to suppress spontaneous EVT differentiation in human

**Figure 7:**
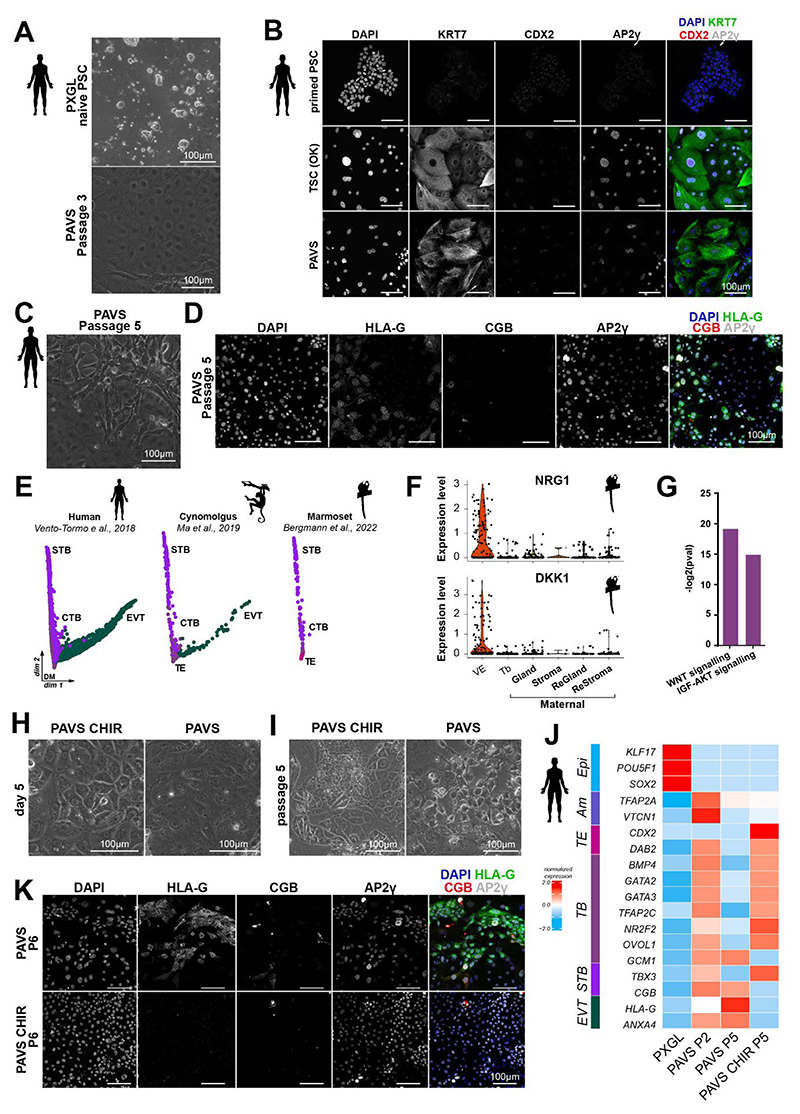
WNT activation prevents EVT differentiation in human PAVS. (A) Phase contrast images of naive human PSCs differentiated in PAVS until passage 3. (B) IF of trophoblast markers in indicated conditions. (C) Phase contrast image of passage 5 human PAVS. (D) IF of markers of trophoblast differentiation in indicated conditions. (E) Cross species diffusion map analysis in human, marmoset and cynomolgus monkey. TE: trophectoderm, CTB: cytotrophoblast, STB: syncytiotrophoblast, EVT: extravillous trophoblast. (F) Violin plot of normalized expression in extraembryonic marmoset lineages and in remodelled and native endometrial tissue. reGland: remodelled glands, reStroma: remodelled stroma. (G) Gene ontology terms of significantly enriched signalling pathways. (H,I) Phase contrast images of naive human PSCs differentiated for (H) 5 days in indicated conditions or (I) at passage 5 of indicated conditions. (J) qPCR of lineage markers in human PSCs and TSCs at different passages. Expression values are normalized by row using Z-score. PXGL: naive PSCs, PAVS: TSCs, P2: passage 2, P5: passage 5, Epi: epiblast, Am: amnion, TE: trophectoderm, TB: general trophoblast markers, STB: syncytiotrophoblast, EVT: extravillous trophoblast. (K) IF of passage 6 trophoblast differentiation markers in indicated conditions. See also Figure S7.

FGF/ERK and TGFβ/NODAL inhibition are integral to human TSC and marmoset periTSC culture conditions. (Figure 3A-C)^27,29^. To test whether the marmoset periTSC culture regime can induce trophoblast identity in human, we cultured naive human PSCs^51,72^ in PAVS conditions. PAVS induced flat, epithelial-like cells within 3 days that could readily be passaged (Figure 7A). Human PAVS colonies expressed KRT7 and AP2γ (*TFAP2C*) (Figure 7B), indicating that PAVS conditions promote a trophoblast-like state in human naive PSCs at early passages. However, after three to five passages, small cell populations gradually acquired a mesenchymal, spindle-shaped morphology (Figure 7C). Lineage marker analysis revealed that the spindle-shaped cells in passage 5 human PAVS cultures expressed the EVT marker HLA-G (Figure 7D). This result suggests that human trophoblast-like PAVS cells gradually differentiate into the EVT lineage.

To determine potential drivers of primate EVT and syncytiotrophoblast formation, we generated a single-cell embryo profiling compendium for the trophoblast lineages of human^9^, cynomolgus monkey^73^ and marmoset^34^. Integrated analysis revealed the formation of two branches, representing syncytiotrophoblast and EVT differentiation (Figure 7E,S7A). Trophectoderm and early cytotrophoblast clustered in close proximity at the root of the syncytiotrophoblast and EVT trajectories in human and cynomolgus. Notably, marmoset samples did not form EVT at the examined stages (CS5-7), demonstrating a species-specific adaptation. This observation is consistent with the reported delayed formation of proliferative trophoblast projections in marmoset compared to human^74^. Our previous experiments suggested that NRG1 is a conserved agonist of EVT differentiation in both marmoset and human (Figure 6). Notably, NRG1 is secreted by decidualised stromal cells in human^75^. In accordance with delayed EVT formation, marmoset decidualised stromal cells did not express NRG1 at CS5-7 (Figure 7F). To further identify species-specific adaptations in cell signalling, we performed signalling pathway analysis between human and marmoset cytotrophoblasts. Gene ontology showed that WNT signalling was significantly enriched in the marmoset trophoblast (Figure 7G), suggesting that endogenous WNT may play a role in preventing premature EVT differentiation. Consistent with this notion, the soluble WNT inhibitor *DKK1* was highly expressed in human decidualised endometrial stroma^76^ but scarcely detectable in marmoset (Figure 7F). These data highlight a potential role for WNT signalling in human EVT differentiation *in vivo*.

WNT activation is a core component of OK medium for TSC self-renewal and is suggested to stabilize the cytotrophoblast state (Haider et al., 2018; Okae et al., 2018; Shannon et al., 2022). We tested if WNT activation with the GSK-3β inhibitor CHIR99021 could prevent EVT differentiation in PAVS conditions (Figure 7H). Naive human PSCs were cultured in PAVS and PAVS plus CHIR99021 (PAVS+CHIR). In both media, naive PSCs flattened out and generated epithelial colonies within three to five days (Figure 7H). By passage 5, PAVS+CHIR samples sustained a TSC-like epithelial morphology, whereas PAVS TSCs differentiated into EVT-like cells (Figure 7C,I). Transcriptional profiling of lineage markers of naive PSCs, PAVS and PAVS CHIR at passage 5 showed that human cells in PAVS downregulated cytotrophoblast markers, including *GATA2, GATA3* and *NR2F2* and showed strong upregulation of EVT markers *HLA-G* and *ANXA4* (Figure 7J). However, PAVS+CHIR robustly expressed *GATA3, GATA2, TFAP2C, NR2F2* and *OVOL1*, consistent with TSC identity (Figure 7J). At the protein level, human PAVS cells showed widespread upregulation of EVT-specific HLA-G and CGB at passage 6 (Figure 7K,S7B). In contrast, PAVS+CHIR cultured human cells did not express trophoblast differentiation markers, maintained epithelial morphology and were positive for AP2γ (*TFAP2C*) (Figure 7K). These results demonstrate that in human, but not marmoset, TSCs require WNT signalling to block EVT differentiation in PAVS conditions.

## Discussion

Here, we report the derivation of marmoset postimplantation- and periimplantation-like TSCs from naive PSCs. Marmoset TSCs exhibited characteristic lineage marker expression, transcriptional correspondence to trophoblast *in vivo*, long-term self-renewal and the ability to differentiate into multinucleated syncytia and EVT. Importantly, marmoset periTSCs readily formed Tb-spheroids that recapitulated trophectoderm-like cell polarity and underwent primary syncytium formation upon attachment *in vitro*. The establishment of marmoset TSCs from naive PSCs corroborates the concept of epiblast plasticity in human^25,27,29^. Future studies will be required to delineate the timing of lineage restriction in human and non-human primate embryos.

Amnion exhibits a remarkable degree of similarity to trophoblast in the primate embryo: both form thin, squamous epithelia, give rise to a central lumen and are controlled by a closely-related transcriptional circuitry^34,58,77–79^. Marmoset peri- and postTSCs demonstrated substantially reduced expression of all amnion markers. Methylome analysis of marmoset TSCs revealed hypermethylation of the amnion marker VTCN1, which may present a potential regulatory mechanism to prevent amnion trans-differentiation. Moreover, another key difference between amnion and trophoblast is cell polarity: trophectoderm has an outer apical side and a basal surface towards the inside of the cavity, while the amnion forms a rosette with an inner apical and outer basal orientation^39,80–82^. We show that Tb-spheroids exhibit trophectoderm-like polarity in the absence of exogenous ECM, in contrast to amnion-like spheroids^56^.

New World monkeys, including the marmoset, exhibit the most shallow modes of trophoblast invasion amongst primate species ^39,40,83^. Thus, marmoset TSCs provide an avenue to capture features of superficial implantation and divergent dynamics of EVT differentiation, compared to human. Elucidating the molecular mechanisms regulating trophoblast invasion depths will be important for our understanding of pathophysiological changes in placental development. Prominent examples include pre-eclampsia, where the trophoblast fails to invade the uterus sufficiently, or placenta accreta spectrum disorders, which are caused by excessive trophoblast invasion ^69^. Marmoset trophoblast expands within the uterine cavity without interstitial or endovascular trophoblast invasion during the first month of development *in vivo* ^39,74,84^. Cross-species transcriptome analysis of human, cynomolgus monkey and marmoset embryo datasets indicated an absence of EVT-like cells in the marmoset at CS5-7, yet marmoset TSCs were capable of EVT lineage acquisition. It is tempting to speculate that the absence of NRG1 expression in the marmoset endometrium may contribute to a later onset of EVT formation *in vivo*. Alternatively, human and marmoset trophoblast cells may differ with regard to endogenous WNT signalling. Marmoset cytotrophoblast was enriched for WNT signalling compared to human and WNT activation was required to prevent EVT differentiation in human, but not marmoset. Future studies will be required to delineate the role of WNT in EVT formation and to determine if the marmoset maternal niche secretes signals to delay trophoblast invasion for superficial implantation.

Collectively, our work presents a proof-of-concept for capturing the evolutionary divergence of primate trophoblast development. The comparative analysis of human and marmoset TSCs will be a powerful framework to elucidate primate placentation, regulatory mechanisms of invasion depth and pathophysiological changes in human placental development.

### Limitations of the study

Profiling of marmoset TSCs derived from marmoset blastocysts or first trimester placenta were not included due to technical limitations. We were not able to perform chimeric integration with marmoset embryos due to embryo scarcity. Matrigel alone is insufficient to faithfully recapitulate the maternal environment. Future studies must incorporate endometrial glands and stromal cells. While we have performed significant validation of *in vivo* marmoset sample identity, the inability to identify cell borders during tissue lysis makes samples with multiple cell types possible.

## Star Methods

### Resource Availability

#### Lead contact

Further information and requests for resources and reagents should be directed to and will be fulfilled by the lead contact, Thorsten Boroviak (teb45@cam.ac.uk).

#### Materials availability

Plasmids and marmoset lines generated in this study are available from the lead contact upon request.

## Experimental Model And Study Participant Details

### Animals

All animal work was carried out according to the Animals (Scientific Procedures) Act 1986 Amendment Regulations 2012 with ethical review by the University of Cambridge Animal Welfare and Ethical Review Body (AWERB).

Marmoset embryo samples were originally obtained in Bergmann et al., 2022^34^ with new specimen analysed in this study. Briefly, marmoset embryos were collected at the German Primate Center (Deutsches Primatenzentrum-Leibniz Institute for Primate Research) according to the German Animal Protection Law and approved by German Primate Center ethics committee. Animals were obtained from self-sustaining marmoset monkey (*C.jacchus*) breeding colony of the German Primate Center and housed according to the standard German Primate Center practice for common marmoset monkeys. Females were aged between 8-11 years old. Animal procedures to retrieve the marmoset embryos used in this study were approved by the Niedersächsisches Landesamt für Verbraucherschutz und Lebensmittelsicherheit, LAVES, under licence number 33.19-42502-04-16/2130 ‘Gewinnung früher Implantationsembryonen des Weißbüschelaffen zur molekularen Charakterisierung frühembryonaler Differenzierungsschritte bei Primaten’, which included a positive ethics statement. Uterus isolation was performed as described in Bergmann et al., 2022 and was carried out by specialised and experience veterinarians.

Mice used in this study were kept according to national and international guidelines in the animal facility of the University of Cambridge. Mouse embryos were obtained from Charles River through natural mating of CD1 wildtype mice and culled at the time of embryo collection by cervical dislocation. Mice used for wildtype embryo matings were 6 weeks old.

### Cell lines

Embryo-derived conventional marmoset PSCs lines New2 (female) and New4 (female) were provided by E. Sasaki. Marmoset PSCs were maintained in KSR/bFGF medium, which is comprised of Dulbecco’s modified Eagle medium (DMEM)/F12 (21331, Gibco) supplemented with 20% Knockout Serum Replacement (KSR) (10828028, Thermo Fisher Scientific), 1% GlutaMAX (35050061, Thermo Fisher Scientific), 1% MEM non-essential amino acids (11140035, Thermo Fisher Scientific), 100 μM β-mercaptoethanol (21985023, Thermo Fisher Scientific) and 10 ng/mL bFGF (Cambridge Stem Cell Institute). Cells were routinely cultured on mitomycin C (M4287, Sigma-Aldrich) inactivated mouse embryonic fibroblast (MEF) feeder cells (Cambridge Stem Cell Institute) under 5% O_2_ and 5% CO_2_ at 37 °C. The medium was changed daily and cells were passaged every 2–4 days by dissociation with Accutase (00-4555-56, Thermo Fisher Scientific) for 5 min.

All human PSCs experiments were approved by the UK Stem Cell Bank Steering Committee and comply with the regulations of the UK Code of Practice for the use of Human Stem Cell Lines. The human embryonic stem cell line SHEF6^85^ was provided by the laboratory of A. Smith with ethical approval from the UK Stem Cell Bank. Conventional SHEF6^86^ were cultured on vitronectin-coated dishes (10 µg/ml; A14700, Thermo Fisher Scientific) in Essential 8 (E8) medium (A1517001, Thermo Fisher Scientific) under hypoxic conditions (37 °C, 5% CO_2_, 5% O_2_). Cells were routinely passaged in clumps using 50 mM EDTA (AM9261, Invitrogen). All stem cells were karyotyped, routinely tested for mycoplasma contamination and authenticated by RNA-seq.

### Method Details

#### Marmoset and human PSC resetting

Marmoset PSCs were reset chemically as previously described in Bergmann et al., 2022^34^. In short, conventional marmoset PSCs were seeded as clumps of 2–5 cells one day before resetting at 50,000 cells per well of a 12-well plate (1.3 × 10^4^ cells per cm^2^) on MEFs. After 24 h, the medium was changed to PLAXA, which comprised N2B27 (Ndiff; Y40002,Takara Bio) medium supplemented with 1% chemically defined lipids (11905031, Gibco), 1 μM PD0325901 (Cambridge Stem Cell Institute), 10 ng/ml recombinant human leukaemia inhibitory factor (LIF; Cambridge Stem Cell Institute), 50 μg/ml L-ascorbic acid (A4403, Sigma-Aldrich), 2 μM XAV939 (SM38-200, Cell Guidance Systems) and 20 ng/ml activin A (Cambridge Stem Cell Institute). Throughout conversion, cells were passaged with Accutase 1:1.5 every 3–4 days. Dome-shaped colonies first emerge at days 4–5 and naive conversion is complete by day 9.

SHEF6 human PSCs were reset chemically using the protocol established by the Smith laboratory^72,87^. Cells were treated with 1 µM PD0325901, 10 ng/ml human LIF and 1 mM valproic acid (VPA; P4543, Sigma-Aldrich) in N2B27 for 3 days on inactivated MEFs. Then, the medium was changed to PXGL: 1 μM PD0325901, 2 μM XAV939, 2 μM Gö6983 (2285, Bio-Techne) and 10 ng/mL human LIF in N2B27. 10 µM Y-27632 (1254, Tocris) was added during initial resetting in some cultures for the first 10 days.

#### Trophoblast stem cells derivation from PSCs

Naive PSCs were prepared as previously described on MEFs and cultured to 60-70% confluency. Naive PSCs media was removed and PSCs were washed in PBS (D8537, Sigma-Aldrich). Media was replaced with indicated culture conditions and differentiated for 5 days.

OK conditions were as previously described in Okae et al., 2018^20^. Other conditions include OK XAV PD: advanced DMEM/F12 (12634010, Thermo Fischer Scientific) supplemented with 55 μM β-mercaptoethanol, 0.3% bovine serum albumin (BSA; A9418, Sigma-Aldrich), 1% ITS-X supplement (41400045, Gibco), 1.5 mg/ml L-ascorbic acid, 50 ng/ml EGF (E9644, Sigma-Aldrich), 2 μM XAV939, 1 μM PD0325901, 0.5 µM A83-01 (72022, Stem cell technologies), 1 µM SB431542 (1614, Tocris), 0.7 mM VPA and 1 µM Y27632 and PAVS: advanced DMEM/F12 supplemented with 0.3% BSA, 1% ITS-X supplement, 55 μM β-mercaptoethanol, 1% chemically defined lipids, 1x GlutaMAX, 1 μM PD0325901, 0.5 µM A83-01, 1µM SB431542, 0.7 mM VPA and 1 µM Y27632. Both conditions were cultured on inactive MEFs unless otherwise specified. 5 µg/ml collagen IV (C5533, Sigma-Aldrich) coating was generated by resuspending collagen in PBS and coating at 37°C for 2 hours. Additional molecules added include 2 μM XAV939, 20 ng/mL BMP4 (314-BP, R&D systems), 2 µM CHIR99021 (CHIR; Cambridge Stem Cell Institute), and 20 μM forskolin (CAY11018, Cambridge Bioscience). Media was changed every 24 hours.

After 4-5 days or at 90% confluency, epithelial colonies were washed with PBS and dissociated with TrypLE (12605010, Thermo Fisher Scientific) for 6-8 minutes or until cells balled up and formed 2-3 cell clumps. TrypLE was carefully removed to not disturb cells and cells were resuspended and replated in culture media supplemented with 10 µM Y-27632 for 24 hours. Marmoset TSCs were passaged 1:2 every 7 days or until 80% confluence was reached.

#### Overexpression marmoset PSC lines generation

Overexpression plasmids were designed in the *Vectorbuilder* vector design tool and were transfected with a pBase plasmid containing the piggybac transposase. LifeACT plasmid is as described in Riedl et al., 2008^88^.

OCT4 OE: pPB[TetOn]-TRE>cm*POU5F1*:BGH pA:CMV>Puro-rev(CAG>tTS: T2A:rtTA)

CDX2 OE: pPB[TetOn]-TRE>cmCDX2:BGH pA:CMV>Neo-rev(CAG>tTS: T2A:rtTA)

NLS-GFP: pPB[Exp]-Puro-CAG>NLS-EGFP

LifeACT-mCherry: pPB[Exp]-Blast-CAG> LifeACT-mCherry

Cerulean: pPB[Exp]-Puro-CAG>Cerulean

Solution 1: 250μL of OptiMEM (31985062, Thermo Fisher Scientific) containing 5 μg of the gene plasmid and 2.5 μg of pBase. Solution 2: 250 μL of OptiMEM with 15 μL of Lipofectamine™ 2000 Transfection Reagent (11668019, Thermo Fisher Scientific). Solutions were incubated separately at room temperature for 5 minutes before being combined and incubated at room temperature for 30 minutes. Marmoset PSCs were cultured in 6-well culture dishes to 75-80% confluency and 2mL of fresh media was placed on top of cells. Combined solution was then evenly distributed drop-wise over the marmoset PSCs. Cells and transfection solutions were incubated overnight. Medium was replaced with fresh culture medium the next day and cells were allowed to recover for 24-48 hours. Culture medium supplemented with either 2.5 μg/mL of puromycin (Cambridge Stem Cell Institute), 1 μg/mL of blasticidin (Cambridge Stem Cell Institute) or 200 μg/mL G418 (MIR 5920, Cambridge Bioscience) for selection of transfected colonies for 48 hours before cells were passaged. Plasmid-specific gene expression of the gene of interested was assayed via qPCR to confirm successful transfection. Dox-inducible gene expression was promoted by supplementing culture medium with 1 μg/mL doxycycline (CAY14422, Cambridge Bioscience) for a minimum of 2 passages.

#### Trophoblast spheroids generation and culture

Cells were encapsulated as previously described ^56,67,68,89^. In short, microfluidic devices were designed with two inlets for aqueous low-melting agarose with cells and a continuous oil phase. Cells were resuspended at 1.5 × 10^6^ cells/100 μL in PBS with 3% BSA. The cell suspension was mixed 1:1 with low-melting-point agarose (50302, Lonza) solution at 37°C. HFE-7500 (Fluorochem) supplemented with surfactant (0.3%; C022, Pico-Surf by Sphere Fluidics) was used as the continuous oil phase. Syringes (SGE Analytical Science) controlled by automated pumps (CETONI, neMYSIS) injected the agarose-cell suspension separately from the oil-surfactant solution into the microfluidic chips for emulsification. Agarose droplets left the microfluidic chip through the outlet and were collected on ice for polymerization. For demulsification, 200 µL medium and 45 µL 1H,1H,2H,2H-perfluoro-1-octanol (B20156.18, AlfaAesar) were used.

Encapsulated marmoset and human TSCs were cultured in indicated medium supplemented with 1% penicillin-streptomycin (15140122, ThermoFisher Scientific), 1% Matrigel for non-invasion assays (354230, Corning) and 10 µM Y-27632. Microgel-suspension cultures were cultured at 37°C under hypoxic conditions (5% CO_2_ and 5% O_2_). Marmoset TSC spheroids were cultured in the presence of MEFs. Media was topped up every other day with twice the amount of media. On alternate days, media was completely changed by spinning microgels down at 200g for 5 minutes. Supernatant was carefully removed, leaving at least 0.5 mL of media per well. Microgels were resuspended and transferred back to the same well. On day 4-5 (depending on structure growth), structures were released from microgels by incubation with 1 U/ml Agarase (EO0461, Thermo Fisher Scientific) in N2B27 medium for 5 min at 37°C, followed by gentle pipetting up and down to free structures from digested agarose.

Hanging drops were generated by gently dissociating cells with TrypLE for 6-8 minutes, ensuring cell clumps remained. TrypLE was removed carefully to not disturb dissociated cells. Cells were gently resuspended in the appropriate TSC media and plated in 10-15 µL droplets on the lid of a 15 cm dish. The bottom of 15 cm dish was filled with distilled water and the lid was carefully flipped. Hanging droplets were cultured for 2-3 days. 10 µL droplets were given 10 µL of fresh media on day 2.

#### Embryo cryosections

Pregnant marmoset uteri were obtained as described in Bergman et al., 2022 and were embedded unfixed into optimum cutting temperature (OCT) compound (4583, TissueTek) and snap-frozen^34^. Each OCT block containing uteri with implanted embryos was sectioned fully at a thickness of 12 μm using a Leica cryostat microtome (CM3050) to obtain consecutive slices of the whole organ. Sections containing embryo tissue were collected either on Naphthalate (PEN) membrane slides (Zeiss, 1.0PEN) or histological slides (Superfrost Plus, Thermo Fisher Scientific) for LCM and immunostaining analysis, respectively, and immediately transferred to dry ice.

#### Cross-species marmoset TSCs and mouse chimera

Zygotes were collected at E0.5 by removing the cumulus cells with brief incubation in hyaluronidase (H4272, Sigma-Aldrich). Embryos were then cultured for 2 days in EmbryoMax Advanced KSOM medium (MMR106D, Sigma-Aldrich) until they reached 8-cell stage. For aggregation chimeras, zona pellucidae were removed from the embryos by brief incubation in acidic Tyrode’s solution (T1788, Sigma-Aldrich) for 30 sec - 1 min at RT.

Marmoset cerulean-tagged periTSCs were washed with PBS and dissociated with TrypLE for 10-15 min. TrypLE was carefully removed and cells were resuspended in N2B27. Clumps of 2-3 cells were rinsed in KSOM (MR-101, Sigma-Aldrich) and manually aggregated with zona pellucida-depleted 8-cell stage mouse embryos in microwells generated with a Hungarian darning needle. Aggregated embryos were then cultured *in vitro* in EmbryoMax Advanced KSOM overlayed with mineral oil for 48h, until E4.5 equivalent. All embryo culture steps were performed in 5% CO_2_ at 37°C.

#### EVT differentiation and migration assay

Marmoset TSCs and human OK cells were differentiated to EVT according to the protocol described in Okae et al., 2018^20^. Medium was changed every 24h for marmoset TSCs. After 6 days of differentiation, cells were further analysed or assayed for migratory properties.

Cells were cultured in 5% CO_2_ and 5% O_2_ at 37°C.

Migration was assessed by the ability of TSCs to cross polyethylene terephthalate membrane transwell inserts (8 μm pore size; 353097, Corning). TSCs were dissociated using TrypLE for 10-15 min and resuspended in the appropriate medium. Media used for control periTSCs, control human TSCs and EVT cells are PAVS (described previously), OK (described in Okae et al., 2018^20^) and EVT medium without Matrigel (advanced DMEM/F12 with 0.1 mM β-mercaptoethanol, 0.3% BSA, 1% ITS-X supplement, 7.5 μM A83-01, 2.5 μM Y27632, 4% KSR as described in Okae et al., 2018^20^, respectively. TSCs were placed in the upper chamber (50 000 cells per insert) and the lower compartment was filled with medium supplemented with 10% FBS. After 24 hours, media in the upper and bottom chambers were replaced with the respective fresh media without and with FBS respectively. The following day, non-migratory cells on the upper surface of the membrane were wiped away with a cotton swab and migratory cells at the bottom of the membrane were fixed with 4% paraformaldehyde (PFA; 15714S, Electron Microscopy Sciences/Thermo Fisher Scientific) for 15 min at room temperature.

#### Trophoblast attachment and invasion assay

Ibidi μ-Slide 8 wells were coated with indicated ECM. Thin collagen IV coating was generated by incubating 5 μg/ml human placental collagen IV in PBS for 2 hours at 37°C. Thin Matrigel coatings were generated by incubating wells with 1% Matrigel in DMEM for 2 hours at 37°C. Thick Matrigel beds were generated by pre-chilling pipette tips and μ-Slide 8 wells to -20°C. 50µL of Matrigel was pipetted into each chilled well, transferred back into their packaging and spun down in a plate spinner for 30s to create a flat bed. Matrigel-coated Ibidi μ-Slide 8 wells were incubated at 37°C for 1 hour.

Structures were allowed to attach to the ECM coating/beds over 24-48 hours. After attachment, media was changed every 24 hours. Implanted structures were fixed in 4% PFA and treated for IF staining as previously described for 2D culture, with special care taken to not disrupt attached structures.

#### Quantificative polymerase chain reaction

RNA extraction was performed using a Total RNA Miniprep Kit (T2010S, Monarch). Complementary DNA was obtained with GoScript Reverse Transcriptase (A5003, Promega). qPCR was performed with SYBR green PCR Master Mix (4309155, Thermo Fisher Scientific) in a StepOnePlus Real-Time PCR machine (Applied Biosystems). Results were normalized to the geometric mean of UBC and ACTB using the dCt method^90^. Primer sequences can be found in supplementary Table S1.

#### Immunocytochemistry

Cryosection slides were thawed at room temperature and fixed for 8 min in 4% PFA/PBS solution. *In vitro* cells were cultured in Ibidi μ-Slide 8-wells (80806, Ibidi) and fixed with 4% PFA in PBS for 10 min at room temperature. Samples were washed three times with PBS and permeabilised with 0.25% Triton X-100 (13444259, Thermo Fisher Scientific) in PBS for 30 min. Slides and *in vitro* cells were blocked in 2% donkey serum (116-4101, Thermo Fisher Scientific), 0.1% BSA, 0.01% Tween-20 (BP337-100, Thermo Fisher Scientific) in PBS for 30 min and incubated with primary antibody solution overnight 4°C. Slides were kept in a humidified chamber for incubation steps. Tile scanning was performed to image the uterine cavity with embryo in its entirety. Tile-scanned images were automatically merged by the acquisition software.

Secondary antibodies supplemented with nuclear-staining DAPI (4′,6-diamidino-2-phenylindole, Sigma-Aldrich) in blocking buffer were applied after washing steps (three times with PBS) and incubated for 60 min at room temperature. Slides were rinsed and mounted using Vectashield mounting medium (H-1200, Vector laboratories) and coverslips (12343138, Thermo Fisher Scientific).

Mouse embryos were fixed with 4% PFA for 15 min at room temperature. They were washed 3 times in PBS supplemented with 3 mg/mL polyvinylpyrolidone (PBS/PVP; PVP10, Sigma-Aldrich) and permeabilised in 0.25% Triton X-100 PBS/PVP for 30 min. Blocking was achieved with PBS containing 2% donkey serum, 0.1% BSA and 0.01% Tween-20 for 60 min. Embryos were incubated with primary antibodies in blocking buffer overnight at 4°C. Subsequently, embryos were washed 3 times in blocking buffer for a minimum of 15 min each time before incubation with the secondary antibodies for 2h at room temperature in the dark.

Fixed transwell membranes were washed 3 times with PBS and permeabilised with 0.25% Triton X-100 in PBS for 30 min. Following a 30 min incubation in blocking solution (2% donkey serum, 0.1% BSA and 0.01% Tween-20), membranes were transferred into blocking solution supplemented with DAPI for 2h at room temperature. Membranes were rinsed 3 times with blocking buffer and mounted using Vectashield mounting medium and coverslips.

Antibody details are listed in Key resources table.

#### Live imaging

Cells were plated in Ibidi μ-Slide 8 wells and given 400 µL of media to enable extended culture. Cells with larger nuclei or multiple nuclei were preferentially imaged. Cells were imaged at 30-minute intervals on a Leica multiphoton SP8 microscope with a 25x water objective using a z-step size of 1 μm. For excitation, 488 nm and 555 nm lasers were used.

#### SEM imaging

SEM imaging was performed by the Cambridge advanced imaging centre. Cells were grown on glass coverslips of 13 mm diameter. Cells were fixed for at least 24h in fixative (2% glutaraldehyde/2% formaldehyde in 0.05 M sodium cacodylate buffer pH 7.4) at 4°C. Coverslips were briefly dipped twice in cold deionised water (DIW) and plunge-frozen by dipping into liquid nitrogen-cooled ethane. Samples were transferred to liquid nitrogen-cooled brass inserts and freeze-dried overnight in a liquid nitrogen-cooled turbo freeze-drier (Quorum K775X).

Sections were imaged in a Verios 460 SEM (FEI/Thermo Fisher Scientific) run at an accelerating voltage of 4 keV and 0.2 nA probe current using the concentric backscatter detector in field-free mode (low magnification) or immersion mode (high resolution).

#### Single cell RNA sequencing

Transcriptomic characterization of pre and postimplantation marmoset trophoblast utilized previously published marmoset *in vivo* single cell RNA sequencing dataset ^34^. Naive derived OK, postTSCs and periTSCs were transferred using glass capillaries into individual tubes containing RLT buffer (1053393, Qiagen) and immediately frozen in dry ice.

Smart-seq2 library preparation was carried out in 96-well format as previously described^91^. Library quality was assessed using the High Sensitivity DNA Analysis Kit (5067-4626, Agilent) on the 2100 Bioanalyzer system (Agilent). Pooled libraries were sequenced on an Illumina NovaSeq platform with a read length of PE 150 bp. Reads were processed as described in Bergmann et al ^34^. Specifically; reads were trimmed of adapter sequences using TrimGalore! (https://github.com/FelixKrueger/TrimGalore) and mapped to the Common marmoset genome (Callithrix jacchus 3.2.1) using STAR^92^ aligner v2.5.4. Only samples with >100,000 mapped reads and mapping efficiency >40% were used for downstream analysis. Gene counts were quantified using FeatureCounts^93^ v1.6.0 using a modified Ensembl gene annotation file (release 91) ^34^. Marmoset samples passing QC were analysed using Seurat^92,93^ v3.1.2. Feature counts were normalised and standardised using the NormalizeData and ScaleData function.

#### DNA methylation

Bulk whole genome bisulfite sequencing was conducted on three replicates of marmoset periTSCs, primed marmoset PSCs and naive marmoset PSCs. Genomic DNA was extracted using a Monarch Genomic DNA Purification Kit (NEB). Bulk bisulfite sequencing was conducted on an Illumina NovaSeq PE150 Sequencing with ~30x coverage by CD Genomics. For bulk methylation data, reads were first trimmed of adapter sequences using Trim Galore! (https://www.bioinformatics.babraham.ac.uk/projects/trim_galore/) and aligned to marmoset genome *C. jacchus* 3.2.1 using Bismark v0.163 (https://www.bioinformatics.babraham.ac.uk/projects/bismark/). The percentage DNA methylation in CpG context and saved as individual bigwig files. Percentage methylation was summarised over various genomic contexts, including over promoter regions, the entire gene body, over exons and introns and at CpG islands and visualised as a line plot or heatmap using Deep Tools v 3.1.3 (https://deeptools.readthedocs.io/en/develop/). Note that CpG Islands were identified using the cpg_lh function of KentUtils (https://github.com/ENCODE-DCC/kentUtils). Differential methylation regions were calculated on incremental 1kb windows using DMRcaller v 1.4.2^94^.

#### Single cell bisulfite-sequencing

Laser capture microdissection was used to isolate tissues at near single-cell resolution in Carnegie Stage 5 (CS5) and CS7 marmoset embryos. RNA was extracted and used in a separate study^34^ while DNA was extracted for bisulfite sequencing (BS-seq) for use in this study, as outlined in Macaulay et al., 2017^95^.

Samples were analysed as outlined in Clark et al., 2017^96^, were first trimmed of adapter sequences using TrimGalore and subsequently aligned to the marmoset genome (Callithrix jacchus 3.2.1) with Bismark (v 0.22.1), using single-end and nondirectional mode. Duplicate reads were removed using bismark_deduplicate, and methylation extraction run on each sample to capture the percent methylation in CpG context. As the per cell coverage for single cell bisulfite sequencing is typically very low, individual bam files were combined to create pseudobulk representations for each tissue. Cell annotations for the individual samples were first assigned based on the transcriptomic annotation^34^ or by location within the embryo. Methylation extraction in CpG context was run on the pseudobulk tissue bam files. For visualisation bedGraphs were converted to bigwig format and visualised in IGV (2.16.2).

## Quantification And Statistical Analysis

### In vivo and in vitro sample sizing

*In vivo* marmoset single-cell RNA sequencing was performed in 2 biological replicates at each developmental stage (CS5, CS6, CS7). *In vitro* OK, postTSCs (OK XAV PD) and periTSCs (PAVS) were generated using New2 and New4 cell lines in two independent rounds. The “N” represented in Figure 3I, 5J, S2G, S3A-B, S5A,J, S6E,I,K,L, S7B represents independent replicates of each experiment. Cross-species chimeras were performed with periTSCs (PAVS) derived from New4 cell line aggregated with 44 embryos collected from 7 different mice.

### Dimensionality reduction, correlation and diffusion map analysis

The input dataset for dimensionality reduction consists of the 10000 most variably expressed genes, as determined by the Seurat function FindVariableFeatures^97^. *In vitro* cultures were jointly analysed using Seurat based on the canonical correlation analysis and mutual nearest neighbour approaches. For visualisation, data from all *in vitro* models were jointly integrated within the marmoset *in vivo* reference datasets^34^ using Seurat, based on Canonical Correlation Analysis (CCA) and mutual nearest neighbour (MNN) approaches. Specifically, FindIntegrationAnchors was run using 10000 features and IntegrateData (with 20 dimensions) was used to calculate corrected gene expression matrices for the three datasets. Datasets were visualised using PCA on the corrected gene expression matrix, with *in vivo* and *in vitro* datasets split to aid interpretation.

Conserved markers for lineages were identified using the FindMarkers function on the 10,000 integration genes. Conserved markers for lineages were identified using the FindMarkers function with cutoffs based on an adjusted p value of < 0.05 and an average log foldchange >0.1 or <-0.1. Marker expression of key markers was visualized using a scatter plot on the first two principal components. Functional analysis was conducted using the Kyoto Encyclopaedia of Genes and Genomes (KEGG) and Reactome databases via the R package^98^. The Seurat function AddModuleScore evaluated the expression levels of genes within a particular signalling pathway, metabolic pathway or a cluster of genes^99^. PCA and UMAP analysis were performed using built-in Seurat functions. Pearson correlation between subgroups of cells were calculated based on corrected gene expression values following integration with Seurat using the inbuilt R function, cor. Cross correlation matrices were visualised as heatmaps using pheatmap 1.0.12. Differential expression analysis was done using the cut-off of FSR < 0.01.

### Cross-species integrative analyses

For cross-species analysis several embryonic and embryonic-model systems were integrated together. Each dataset for integration contained both an embryonic and extraembryonic component and included:

1. Cynomolgus samples from *in vitro* cultured embryos from day 9 to 20 using Smart-Seq2^73^.
2. Cynomolgus samples of *in vitro* cultured embryos from day 10 -14 using 10X^78^.
3. Marmoset samples of *in vivo* embryos from CS5-7 using SS2 ^34^.
4. Human *in vitro* cultured embryos using SS2 from days 8 – 14^77^.
5. Human 10X samples of 1^st^ trimester trophoblast ^9^.
6. Two blastoid models^100,101^ using SS2 and 10X respectively and one 10X amnioid model^102^.

Datasets were integrated in Seurat v3.2.0^97,103^ using IntegrateData on 5000 marker genes (FindIntegrationMarkers) and 20 principal components (PCs). Once integrated, dimensionality reduction was run based on corrected gene expression matrices. For UMAP, dimensionality reduction was based on the first 20 PCs. Clustering was generated using the FindClusters function. For all annotated datasets, preliminary alignments separated out embryonic from extraembryonic cell lineages into separate clusters that separated well using PCA and UMAP. For trajectory inference, clusters associated with the TE (Cl 1 and 9), trophoblast (Clusters 0,2-7,10-11,13-16,18-20) and amnion (Clusters 6,8,9,12,17) were selected for datasets 1-5 (by subsetting clusters X, Y, Z) and a refined clustering was generated. Any cell lineages with <10 cells were filtered out. Lineages for datasets with available annotations remained unchanged and lineages in unannotated or partially unannotated datasets were assigned lineages based on the dominant cell type in that cluster in other (annotated) datasets. Finally, diffusion maps were generated using destiny v 2.12.0^104^. Three diffusion maps were generated to visualise dynamics:

1. Divergence between syncytiotrophoblast and EVT from CTB dominated the first few diffusion components with amnion and TE sitting close to CTB.
2. A subsequent Diffusion map was generated for the TE, CTB and amnion was generated to better visualise the divergence between these lineages.
3. A final DM focussed on the divergence between CTB and the syncytiotrophoblast and EVT lineages

### Image analysis

IF images were analysed using the open-source software Fiji^105^ to extract signal intensities, circularity and lumen size. DAPI was used to generate a nuclei segmentation mask. EZRIN and F-actin were used for structure and lumen segmentation. For syncytium quantifications, ZO1 and DAPI were used to define cell boundaries and nuclei count, respectively. Individual cells were segmented and quantified individually. In figure 5K, CDX2+ cells were determined by 2 times the standard deviation of CDX2 expression in PSCs.

Immunofluorescence analysis was performed in R. Two-tailed Mann–Whitney test was used to compare between two means for samples that were not normally distributed and Kruskal–Wallis followed by Dunn's multiple comparison test was used to compare between more than two means. All quantifications were performed on three independent biological replicates, except for Figure 5K and S2G with 2 biological replicates. Significance was determined by a p value < 0.05 as indicated by * in the figures and figure captions.

## Supplementary Material

Supplementary

Supplemental Information

## Data Availability

RNA sequencing data have been deposited at ArrayExpress and are publicly available as of the date of publication. Accession numbers are listed in the key resources table. This paper analyses existing, publicly available data. These accession numbers for the datasets are listed in the key resources table. Any additional data reported in this paper will be shared by the lead contact upon request. All original code has been deposited at https://github.com/Boroviak-Lab/Trophoblast and is publicly available as of the date of publication. DOIs are listed in the key resources table. Any additional information required to reanalyze the data reported in this paper is available from the lead contact upon request.
