# Supplementary material for: Marmoset and human trophoblast stem cells differ in signalling requirements and recapitulate divergent modes of trophoblast invasion
Source: Cell Stem Cell. Author manuscript; Available in PMC 2024 Oct 16. (PMC7616712; doi:10.1016/j.stem.2024.09.004)
Supplement: Supplementary [file EMS199345-supplement-Supplementary_.pdf]

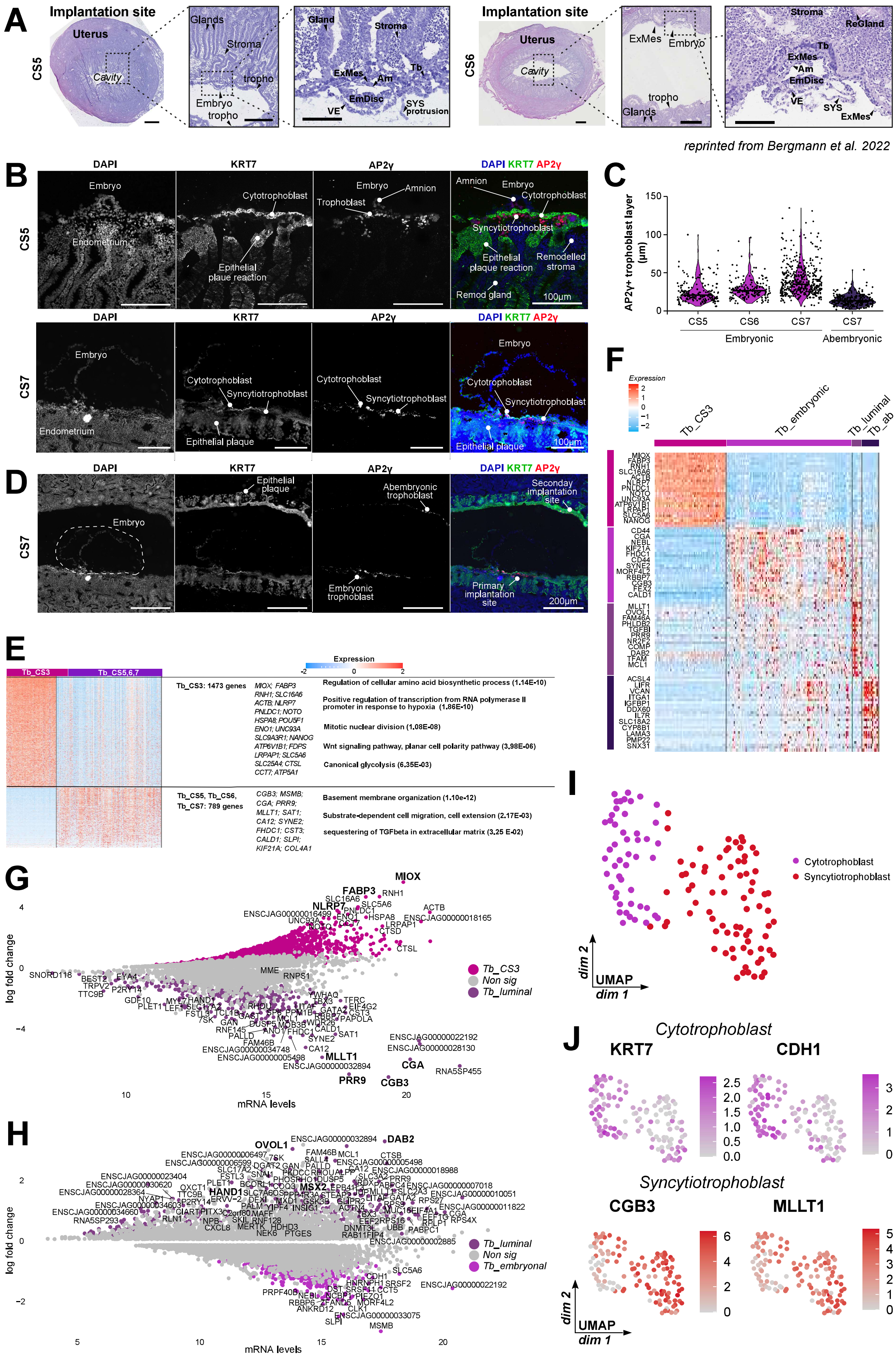

A
CS5
Implantation site
Uterus
Cavity
Glands
Stroma
tropho
Embryo
Gland
ExMes
Am
Tb
EmDisc
VE
SYS
protrusion
CS6
ExMes
Embryo
Glands
ReGland
reprinted from Bergmann et al. 2022
B
DAPI
KRT7
AP2γ
DAPI KRT7 AP2γ
Embryo
Endometrium
Cytotrophoblast
Epithelial plaque reaction
Amnion
Trophoblast
Syncytiotrophoblast
Remodelled stroma
Remod gland
100µm
CS7
Epithelial plaque
C
AP2γ+ trophoblast layer (µm)
CS5
CS6
CS7
Embryonic
Abembryonic
D
Abembryonic trophoblast
Seconday implantation site
Embryonic trophoblast
Primary implantation site
200µm
E
Expression
Tb_CS3
Tb_CS5,6,7
Tb_CS3: 1473 genes
Regulation of cellular amino acid biosynthetic process (1.14E-10)
Positive regulation of transcription from RNA polymerase II promoter in response to hypoxia (1.86E-10)
Mitotic nuclear division (1.08E-08)
Wnt signaling pathway, planar cell polarity pathway (3.98E-06)
Canonical glycolysis (6.35E-03)
Tb_CS5, Tb_CS6, Tb_CS7: 789 genes
Basement membrane organization (1.10e-12)
Substrate-dependent cell migration, cell extension (2.17E-03)
sequestering of TGFbeta in extracellular matrix (3.25 E-02)
F
Expression
Tb_CS3
Tb_embryonic
Tb_luminal
Tb_ab
G
log fold change
mRNA levels
Tb_CS3
Non sig
Tb_luminal
MIOX
FABP3
NLRP7
MLLT1
CGA
PRR9
CGB3
H
log fold change
mRNA levels
Tb_luminal
Non sig
Tb_embryonal
OVOL1
DAB2
HAND1
MSX2
I
Cytotrophoblast
Syncytiotrophoblast
UMAP
dim 1
dim 2
J
Cytotrophoblast
KRT7
CDH1
Syncytiotrophoblast
CGB3
MLLT1

Figure S1. Marmoset trophoblast pre- to postimplantation development, related to Figure 1.

(A) Haemotoxylin and eosin staining of Carnegie stage 5 and 6 marmoset embryos. Reprinted from Bergmann et al., 2022 [1] with permission. Scale bars are 1000 μm (left), 500 μm (middle) and 100 μm (right). (B) IF stainings of CS5 and CS7 marmoset implantation. (C) Thickness quantification of AP2γ+ trophoblast layer in marmoset embryos by stereological confocal microscopy. Each dot corresponds to one measurements. Several measurements were performed per section. Median: CS5 = 20.5 μm , CS6 = 26 μm, CS7 embryonic = 35 μm, CS7 abembryonic = 13.5 μm. (D) IF of CS5 and CS7 marmoset implantation. (E) Heatmap of DEGs between pre- (Tb_CS3) and postimplantation (Tb_CS5,6,7) trophoblast with enriched gene ontological terms. (F) Heatmap of normalized expression of DEGs between trophoblast lineages. (G) MA plot of DEGs between trophectoderm (Tb_CS3) and luminal trophoblast (Tb_luminal). (H) MA plot of DEGs between luminal trophoblast (Tb_luminal) and embryonic trophoblast (Tb_embryonic). (I,J) UMAP of (I) unbiased hierarchical clustering of embryonic trophoblast with (J) normalized log expression of cytotrophoblast and syncytiotrophoblast lineage markers.

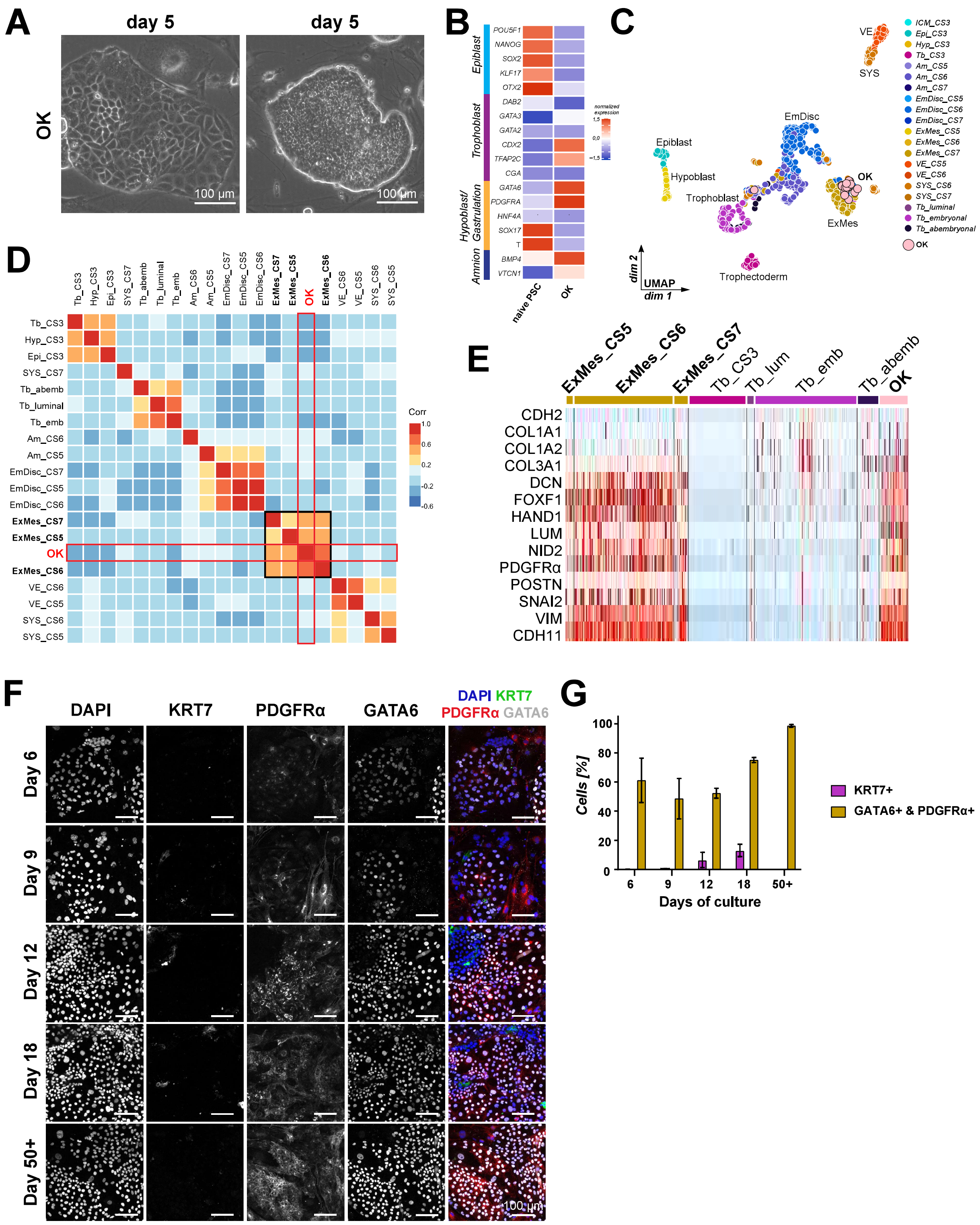
A
day 5
day 5
OK
100 µm
100 µm
B
Epiblast
Trophoblast
Hypoblast/ Gastrulation
Amnion
POU5F1
NANOG
SOX2
KLF17
OTX2
DAB2
GATA3
GATA2
CDX2
TFAP2C
CGA
GATA6
PDGFRA
HNF4A
SOX17
T
BMP4
VTCN1
naive PSC
OK
C
VE
SYS
EmDisc
Epiblast
Hypoblast
Trophoblast
OK
ExMes
Trophectoderm
UMAP
dim 1
dim 2
ICM_CS3
Epi_CS3
Hyp_CS3
Tb_CS3
Am_CS5
Am_CS6
Am_CS7
EmDisc_CS5
EmDisc_CS6
EmDisc_CS7
ExMes_CS5
ExMes_CS6
ExMes_CS7
VE_CS5
VE_CS6
SYS_CS6
SYS_CS7
Tb_luminal
Tb_embryonal
Tb_abembryonal
OK
D
Tb_CS3
Hyp_CS3
Epi_CS3
SYS_CS7
Tb_abemb
Tb_luminal
Tb_emb
Am_CS6
Am_CS5
EmDisc_CS7
EmDisc_CS5
EmDisc_CS6
ExMes_CS7
ExMes_CS5
OK
ExMes_CS6
VE_CS6
VE_CS5
SYS_CS6
SYS_CS5
Corr
1.0
0.6
0.2
-0.2
-0.6
E
ExMes_CS5
ExMes_CS6
ExMes_CS7
Tb_CS3
Tb_lum
Tb_emb
Tb_abemb
OK
CDH2
COL1A1
COL1A2
COL3A1
DCN
FOXF1
HAND1
LUM
NID2
PDGFRα
POSTN
SNAI2
VIM
CDH11
F
DAPI
KRT7
PDGFRα
GATA6
DAPI KRT7
PDGFRα GATA6
Day 6
Day 9
Day 12
Day 18
Day 50+
100 µm
G
Cells [%]
100
80
60
40
20
0
6
9
12
18
50+
Days of culture
KRT7+
GATA6+ & PDGFRα+

Figure S2. Marmoset naïve PSCs differentiate to ExMes in human trophoblast conditions, related to Figure 2.

(A) Phase contrast images of two predominant epithelial morphologies at day 5 of OK differentiated cells. (B) qPCR of lineage markers in marmoset naive PSCs and OK cells. Expression values are normalized by row using Z-score. (C) UMAP plot of *in vivo* marmoset dataset and *in vitro* cells. EPI=preimplantation epiblast, HYPO=hypoblast, EmDisc=embryonic disc, VE=visceral endoderm. (D) Heatmap of Spearman's correlation between *in vivo* and *in vitro* lineages. (E) Heatmap of sample normalized mRNA expression from *in vivo* and *in vitro* RNA sequencing datasets of ExMes-specific genes. (F) IF of trophoblast and ExMes marker expression during OK differentiation time course from naive PSCs. N=2 (G) Quantification of KRT7+ and GATA6+/ PDGFRα+ cell populations from OK naive differentiation time course shown in (F). Error bars represent standard deviation.

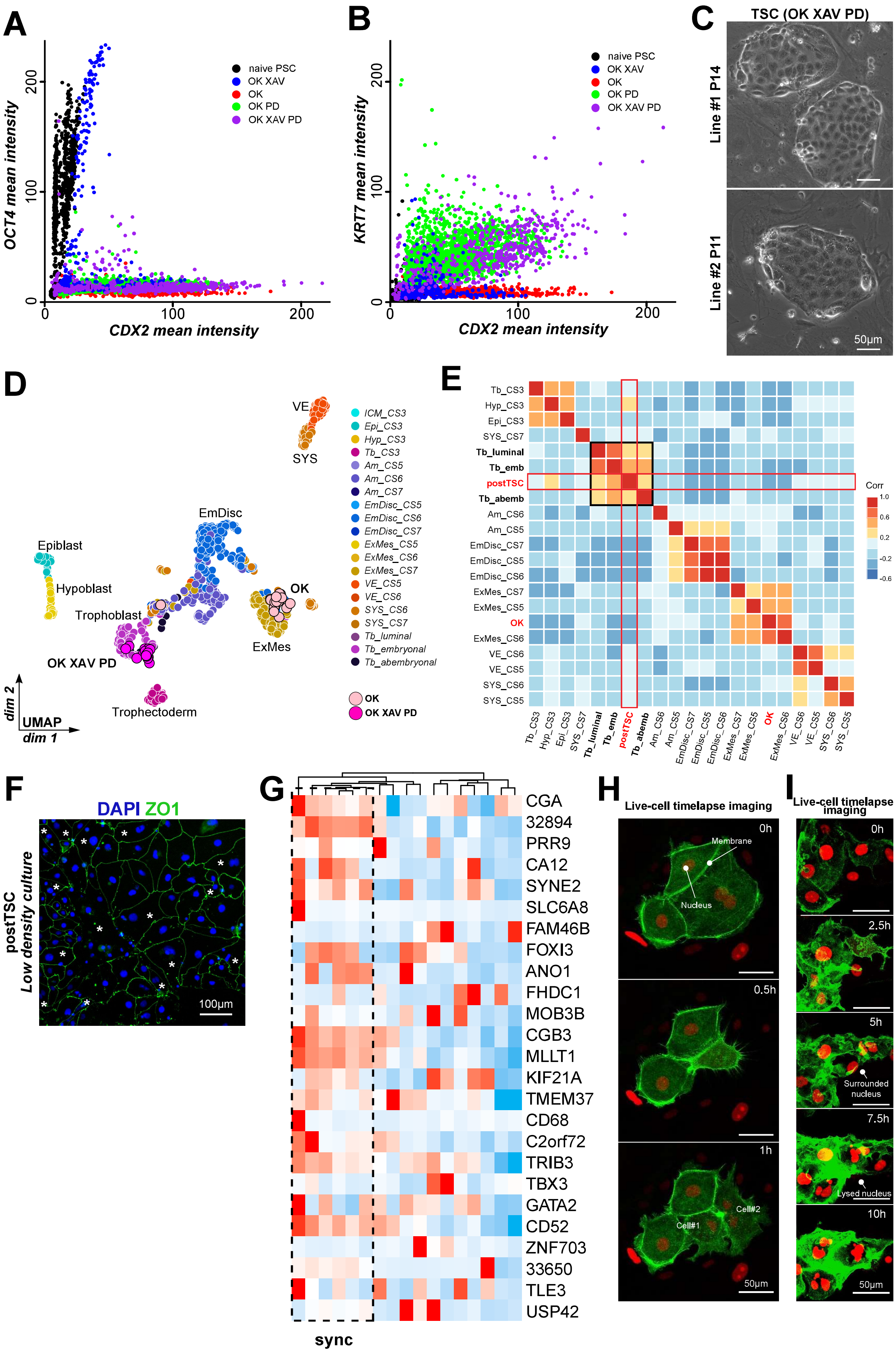
A
OCT4 mean intensity
CDX2 mean intensity
naive PSC
OK XAV
OK
OK PD
OK XAV PD
B
KRT7 mean intensity
CDX2 mean intensity
C
TSC (OK XAV PD)
Line #1 P14
Line #2 P11
50µm
D
VE
SYS
EmDisc
Epiblast
Hypoblast
Trophoblast
OK
ExMes
OK XAV PD
Trophectoderm
UMAP
dim 1
dim 2
ICM_CS3
Epi_CS3
Hyp_CS3
Tb_CS3
Am_CS5
Am_CS6
Am_CS7
EmDisc_CS5
EmDisc_CS6
EmDisc_CS7
ExMes_CS5
ExMes_CS6
ExMes_CS7
VE_CS5
VE_CS6
SYS_CS6
SYS_CS7
Tb_luminal
Tb_embryonal
Tb_abembryonal
E
Tb_CS3
Hyp_CS3
Epi_CS3
SYS_CS7
Tb_luminal
Tb_emb
postTSC
Tb_abemb
Am_CS6
Am_CS5
EmDisc_CS7
EmDisc_CS5
EmDisc_CS6
ExMes_CS7
ExMes_CS5
OK
ExMes_CS6
VE_CS6
VE_CS5
SYS_CS6
SYS_CS5
Corr
F
DAPI ZO1
postTSC
Low density culture
100µm
G
CGA
32894
PRR9
CA12
SYNE2
SLC6A8
FAM46B
FOXI3
ANO1
FHDC1
MOB3B
CGB3
MLLT1
KIF21A
TMEM37
CD68
C2orf72
TRIB3
TBX3
GATA2
CD52
ZNF703
33650
TLE3
USP42
sync
H
Live-cell timelapse imaging
0h
Membrane
Nucleus
0.5h
1h
Cell#1
Cell#2
50µm
I
Live-cell timelapse imaging
0h
2.5h
5h
Surrounded nucleus
7.5h
Lysed nucleus
10h
50µm

Figure S3. Postimplantation marmoset TSCs derived from naive PSCs, related to Figure 3.

(A,B) Quantification of mean intensity of (A) OCT4 or (B) KRT7 with CDX2 in indicated conditions. Each point represents a single cell. N=3. (C) Phase contrast image of passage 15 postTSCs derived in two cell lines. (D) UMAP plot of *in vivo* marmoset dataset and *in vitro* cells. EPI=preimplantation epiblast, HYPO=hypoblast, EmDisc=embryonic disc, VE=visceral endoderm. (E) Heatmap of spearman correlation between *in vivo* and *in vitro* lineages. (F) Representative image of syncytiotrophoblast counting strategy in low density cultures. * indicate counted multinucleated cells. (G) Heatmap of sample normalized mRNA expression from postTSC RNA sequencing datasets of syncytiotrophoblast specific genes. (H,I) Live IF imaging of TSCs tagged with GFP with a nuclear localisation tag (red) and stained with F-actin binding LifeACT (green).

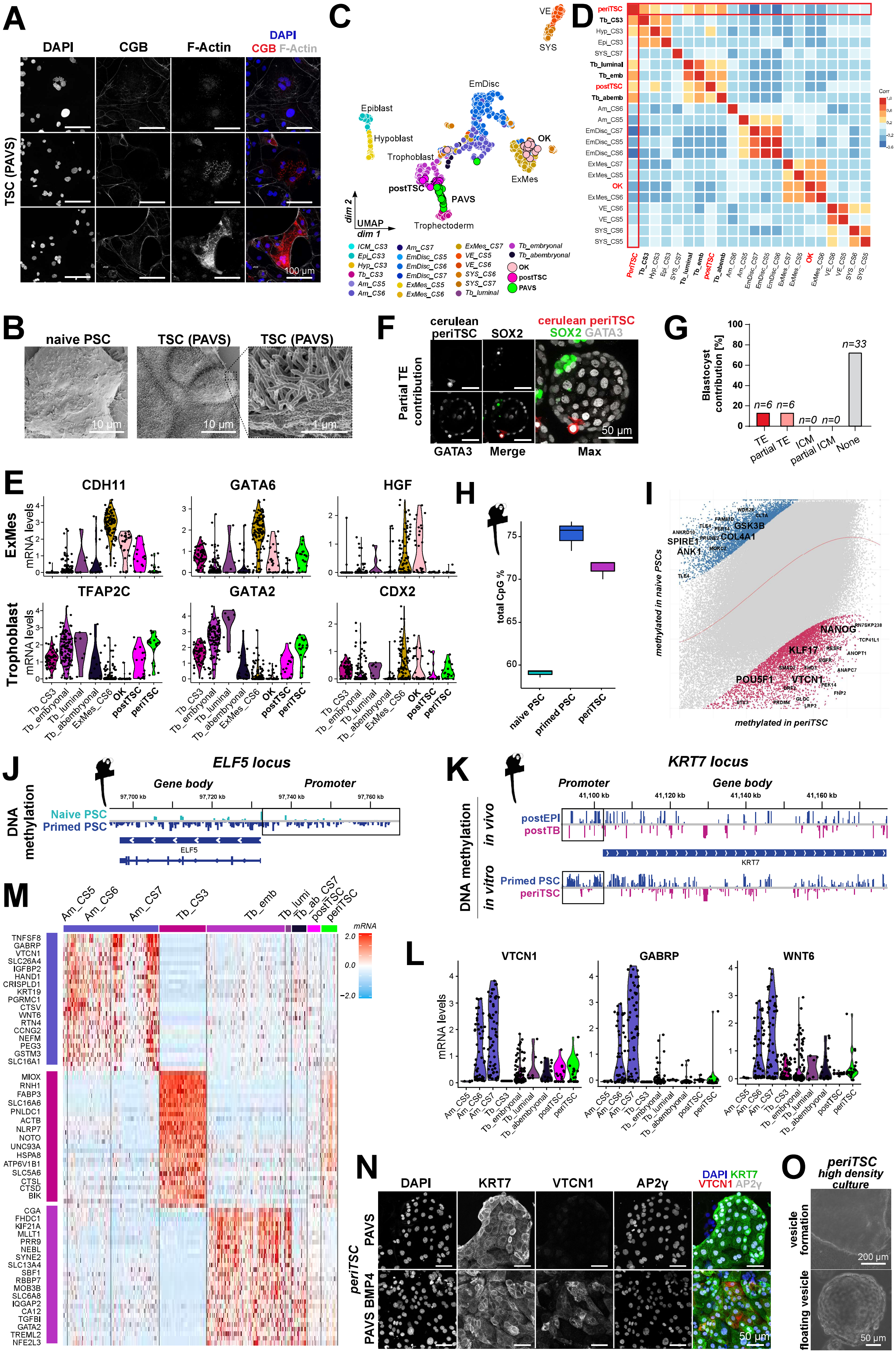
A
DAPI
CGB
F-Actin
DAPI CGB F-Actin
TSC (PAVS)
100 µm
B
naive PSC
TSC (PAVS)
TSC (PAVS)
10 µm
10 µm
1 µm
C
Epiblast
Hypoblast
Trophoblast
EmDisc
OK
ExMes
postTSC
PAVS
Trophectoderm
VE
SYS
UMAP
dim 1
dim 2
D
E
ExMes
Trophoblast
CDH11
GATA6
HGF
TFAP2C
GATA2
CDX2
mRNA levels
F
cerulean periTSC
SOX2
cerulean periTSC SOX2 GATA3
Partial TE contribution
GATA3
Merge
Max
50 µm
G
Blastocyst contribution [%]
n=6
n=6
n=0
n=0
n=33
TE
partial TE
ICM
partial ICM
None
H
total CpG %
naive PSC
primed PSC
periTSC
I
methylated in naive PSCs
methylated in periTSC
GSK3B
COL4A1
SPIRE1
ANK1
NANOG
KLF17
POU5F1
VTCN1
J
DNA methylation
ELF5 locus
Gene body
Promoter
Naive PSC
Primed PSC
ELF5
K
KRT7 locus
Promoter
Gene body
DNA methylation
in vivo
in vitro
postEPI
postTB
Primed PSC
periTSC
KRT7
L
VTCN1
GABRP
WNT6
mRNA levels
M
Am_CS5
Am_CS6
Am_CS7
Tb_CS3
Tb_emb
Tb_lumi
Tb_ab_CS7
postTSC
periTSC
mRNA
N
DAPI
KRT7
VTCN1
AP2γ
DAPI KRT7 VTCN1 AP2γ
periTSC
PAVS
PAVS BMP4
50 µm
O
periTSC high density culture
vesicle formation
floating vesicle
200 µm
50 µm

Figure S4. Periimplantation marmoset TSCs differentiated from naive PSCs, related to Figure 4.

(A) IF of multinucleated cells in periTSC cultures for the syncytiotrophoblast marker CGB. (B) Scanning electron microscopy of marmoset naive PSCs and periTSCs. (C) UMAP plot of *in vivo* marmoset dataset and *in vitro* cells. EPI=preimplantation epiblast, HYPO=hypoblast, EmDisc=embryonic disc, VE=visceral endoderm. (D) Heatmap of Spearman's correlation between *in vivo* and *in vitro* lineages. (E) Violin plot of normalized expression in *in vivo* and *in vitro* lineages of ExMes and general trophoblast markers. (F) IF of E4.5 cross-species aggregation chimaeras with cerulean-tagged periTSCs and mouse embryos demonstrating partial TE contribution. Partially integrated periTSCs showed cytoplasmic contribution to the trophectoderm but lacked correct nuclear alignment. (G) Quantification of lineage contribution of cerulean-tagged periTSCs in chimera embryos (n=44). Embryos were collected from 7 different mice. (H) Global CpG methylation in marmoset *in vitro* cell lines. (I) Differentially methylated regions between marmoset periTSCs and naive marmoset PSCs. (J) Relative methylation of the ELF5 locus in naive and primed PSCs. (K) Relative methylation of the KRT7 locus *in vivo* postimplantation epiblast and trophoblast and *in vitro* primed PSCs and periTSCs. (L) Violin plot of normalized mRNA expression from *in vivo* RNA sequencing datasets of amnion-specific genes. (M) Heatmap of sample normalized mRNA expression from *in vivo* and *in vitro* RNA sequencing datasets of lineage specific genes. (N) IF of trophoblast and amnion (VTCN1) markers in periTSCs treated with or without BMP4. (O) Phase contrast image of periTSCs spontaneously forming spheroids in 2D culture.

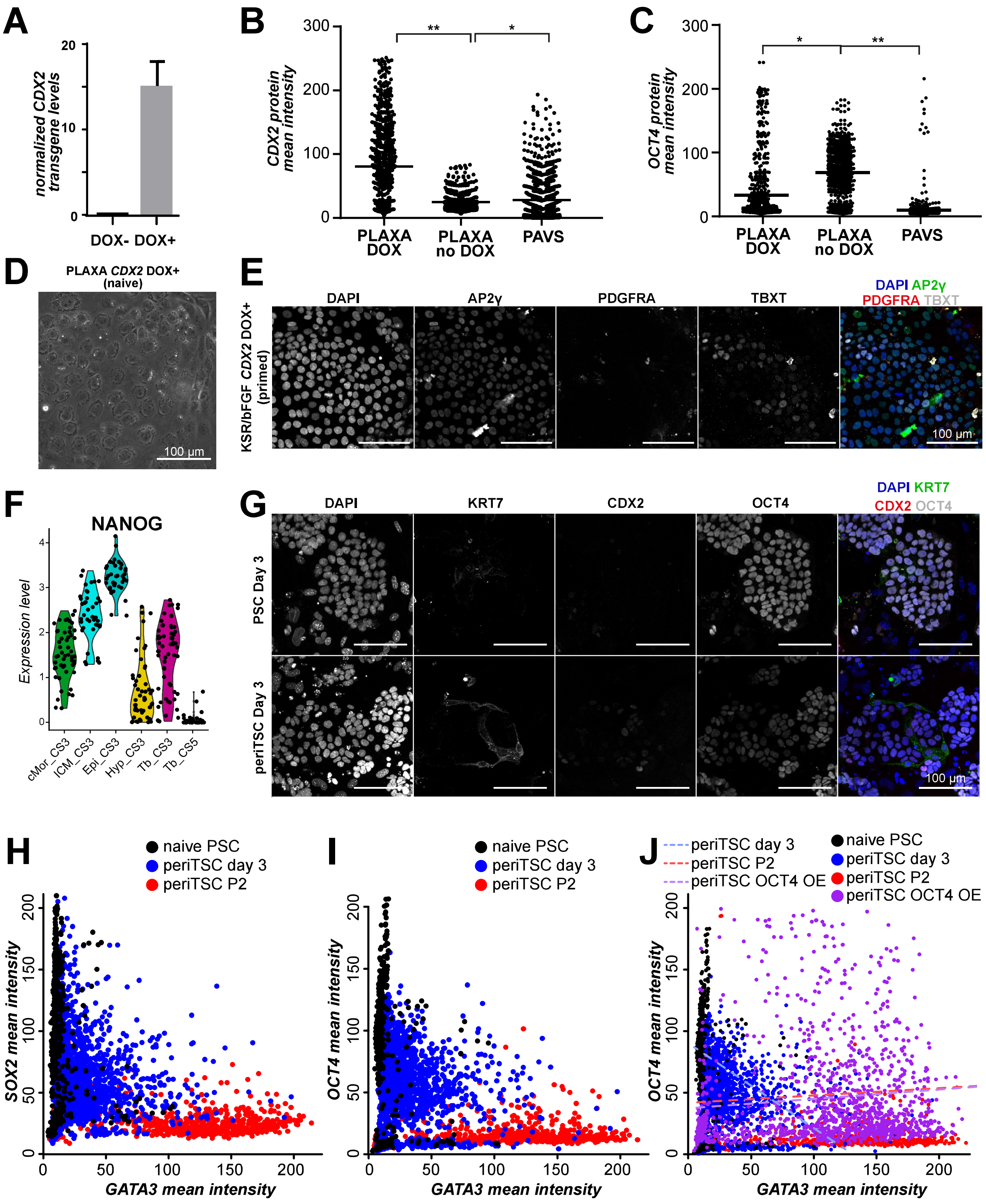
A
normalized CDX2 transgene levels
DOX- DOX+
B
CDX2 protein mean intensity
PLAXA DOX
PLAXA no DOX
PAVS
C
OCT4 protein mean intensity
PLAXA DOX
PLAXA no DOX
PAVS
D
PLAXA CDX2 DOX+ (naive)
100 μm
E
KSR/bFGF CDX2 DOX+ (primed)
DAPI
AP2γ
PDGFRA
TBXT
DAPI AP2γ PDGFRA TBXT
100 μm
F
NANOG
Expression level
cMor_CS3
ICM_CS3
Epi_CS3
Hyp_CS3
Tb_CS3
Tb_CS5
G
DAPI
KRT7
CDX2
OCT4
DAPI KRT7 CDX2 OCT4
PSC Day 3
periTSC Day 3
100 μm
H
naive PSC
periTSC day 3
periTSC P2
SOX2 mean intensity
GATA3 mean intensity
I
naive PSC
periTSC day 3
periTSC P2
OCT4 mean intensity
GATA3 mean intensity
J
periTSC day 3
periTSC P2
periTSC OCT4 OE
naive PSC
periTSC day 3
periTSC P2
periTSC OCT4 OE
OCT4 mean intensity
GATA3 mean intensity

Figure S5. CDX2 is sufficient to promote trophoblast identity in marmoset naïve PSCs, related to Figure 5.

(A) qPCR of CDX2 overexpression mRNA in marmoset PSCs. Overexpression was maintained for 2 passages. (B,C) Quantification of (B) CDX2 or (C) OCT4 mean intensity in CDX2 overexpressing PSCs grown in indicated conditions for 2 passages. Each point represents a single cell. N=2. Significance was calculated using a two-tailed Mann–Whitney test. Error bars represent mean + SD (* - $p<0.05$, ** - $p<0.01$, ***- $p<0.001$). (D) Phase contrast of CDX2 overexpressing epithelial cells in naive maintenance media (PLAXA) with DOX. (E) IF of CDX2 overexpression in primed PSCs for indicated markers. Significance was calculated using a two-tailed Mann–Whitney test. Error bars represent mean + SD (* - $p<0.05$, ** - $p<0.01$, ***- $p<0.001$). (F) Violin plot of normalized expression of pluripotency factors in preimplantation marmoset lineages. (G) IF of PAVS differentiation from naive PSCs at day 3. (H-J) Quantification of mean intensity of indicated proteins in indicated conditions. Overexpression was maintained for 2 passages. Each point represents a single cell. (J) Correlations are indicated as the line of best fit with values as follows: periTSC OCT4 OE (periTSCs OCT4 overexpression)= 0.1065753, periTSCs day 3 = 0.04836971, periTSCs P2 = -0.1520554. Overexpression was maintained for 2 passages. N=2.

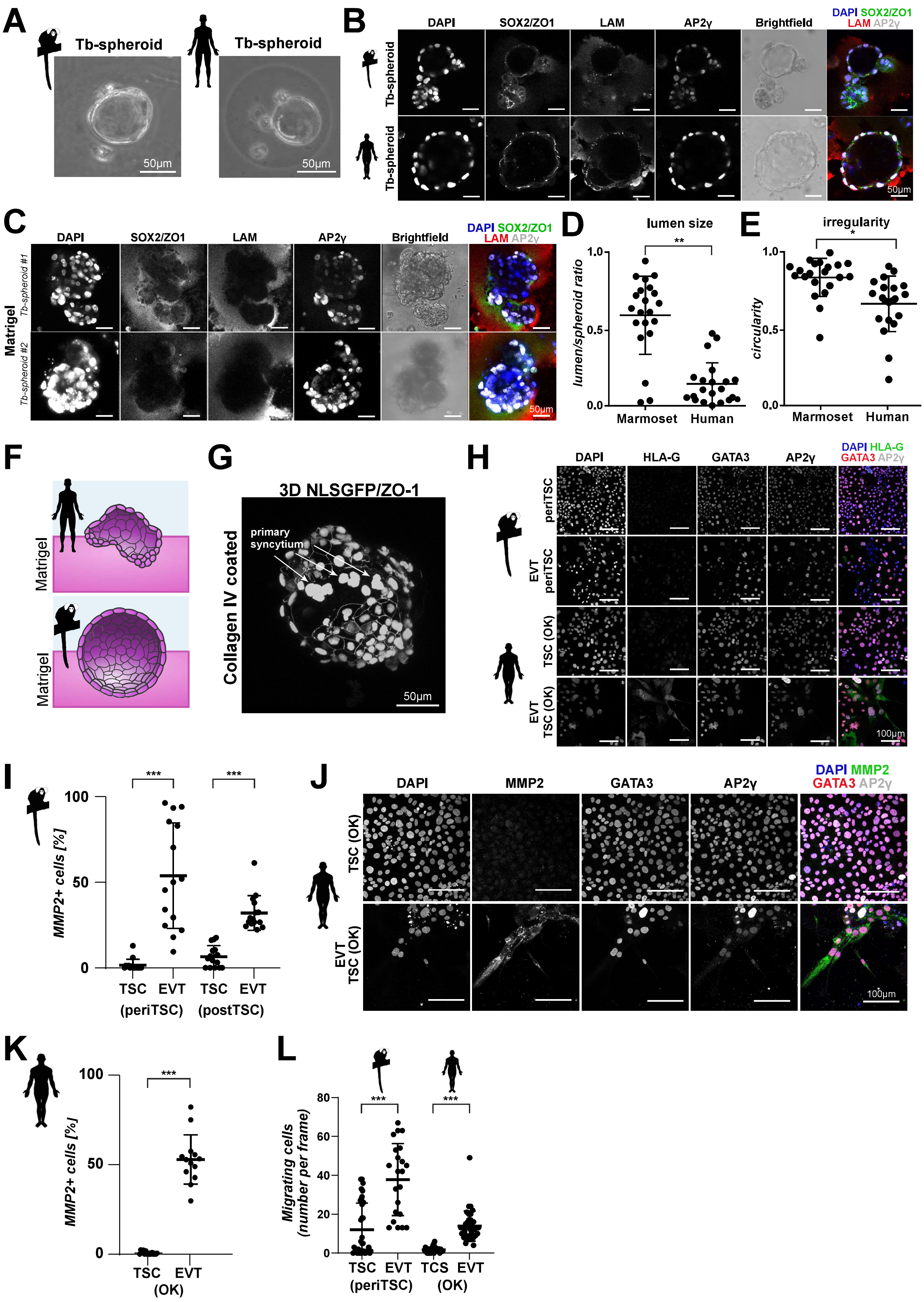

A
Tb-spheroid
Tb-spheroid
50µm
50µm
B
DAPI
SOX2/ZO1
LAM
AP2γ
Brightfield
DAPI SOX2/ZO1 LAM AP2γ
Tb-spheroid
Tb-spheroid
50µm
C
DAPI
SOX2/ZO1
LAM
AP2γ
Brightfield
DAPI SOX2/ZO1 LAM AP2γ
Matrigel
Tb-spheroid #1
Tb-spheroid #2
50µm
D
lumen size
**
lumen/spheroid ratio
Marmoset
Human
E
irregularity
*
circularity
Marmoset
Human
F
Matrigel
Matrigel
G
3D NLSGFP/ZO-1
Collagen IV coated
primary syncytium
50µm
H
DAPI
HLA-G
GATA3
AP2γ
DAPI HLA-G GATA3 AP2γ
periTSC
EVT periTSC
TSC (OK)
EVT TSC (OK)
100µm
I
MMP2+ cells [%]
***
***
TSC
EVT
TSC
EVT
(periTSC)
(postTSC)
J
DAPI
MMP2
GATA3
AP2γ
DAPI MMP2 GATA3 AP2γ
TSC (OK)
EVT TSC (OK)
100µm
K
MMP2+ cells [%]
***
TSC
EVT
(OK)
L
Migrating cells (number per frame)
***
***
TSC
EVT
TCS
EVT
(periTSC)
(OK)

Figure S6. Marmoset TSCs recapitulate postimplantation trophoblast differentiation potential, related to Figure 6.

(A) Phase contrast images of encapsulated marmoset and human TSCs. (B) IF of human and marmoset Tb-spheroids generation with agarose encapsulation cultured with 1% Matrigel. LAM: laminin. (C) IF of embedded human Tb-spheroids on Matrigel beds. LAM: laminin. (D,E) Quantification of (D) lumen size and (E) circularity of attaching human and marmoset spheroids. Each point represents a single structure. N=3. (F) Schematic summary of human versus marmoset attachment assay results. (G) Maximum 3D projection of NLS GFP/ZO-1 channel of a marmoset Tb-spheroid implanting on a thin COLIV coating. (H) IF of control and EVT-differentiated marmoset periTSCs and human TSCs for trophoblast and human EVT markers (HLA-G). (I) Quantification of MMP2+ in periTSCs and postTSCs cultured for 6 days in human EVT conditions. Each point represents a single cell. N=3. (J) IF of human TSCs for the trophoblast (GATA3 and AP2γ) and EVT (MMP2) markers. (K) Quantification of MMP2+ cells in (J). Each point represents a single cell. N=3. (L) Quantification of transwell EVT migration assay. Each point corresponds to the number of migratory cells in one frame. N=3. (D, E, I, K, L) Significance was calculated using a two-tailed Mann–Whitney test. Error bars represent mean + SD (* - $p<0.05$, ** - $p<0.01$, ***- $p<0.001$).

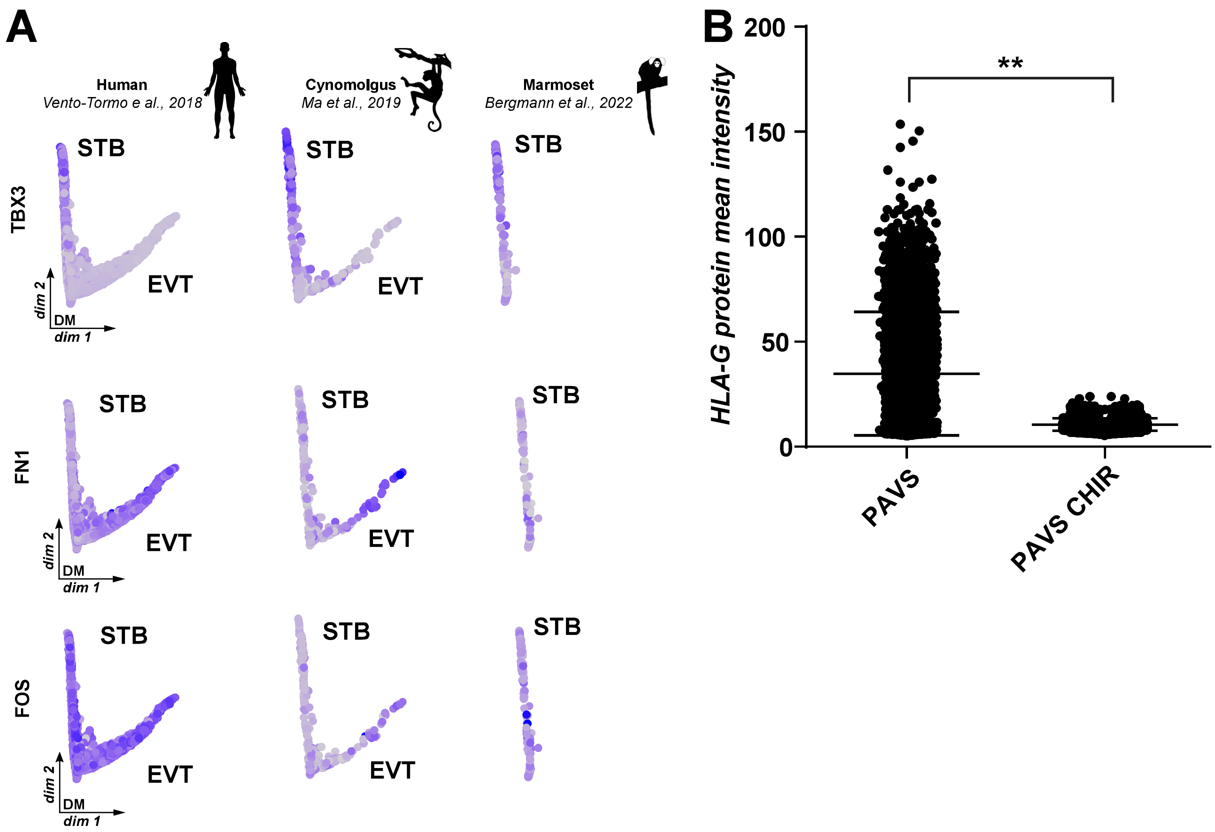
A
Human
Vento-Tormo e al., 2018
Cynomolgus
Ma et al., 2019
Marmoset
Bergmann et al., 2022
TBX3
FN1
FOS
STB
EVT
DM
dim 1
dim 2
B
HLA-G protein mean intensity
0
50
100
150
200
**
PAVS
PAVS CHIR

Figure S7. Diverging implantation strategies between primates, related to Figure 7.

(A) Diffusion maps of cross-species datasets showing normalized log expression of syncytiotrophoblast and extravillous trophoblast markers. STB: syncytiotrophoblast, EVT: extravillous trophoblast. (B) Quantification of HLA-G mean intensity in indicated conditions. Significance was calculated using a two-tailed Mann–Whitney test. N=3. Error bars represent mean + SD (* - $p<0.05$, ** - $p<0.01$).

Supplemental references:

1. Bergmann, S., Penfold, C.A., Slatery, E., Siriwardena, D., Drummer, C., Clark, S., Strawbridge, S.E., Kishimoto, K., Vickers, A., Tewary, M., et al. (2022). Spatial profiling of early primate gastrulation in utero. Nature *609*, 136–143. https://doi.org/10.1038/S41586-022-04953-1.
