# Supplementary material for: Marmoset and human trophoblast stem cells differ in signalling requirements and recapitulate divergent modes of trophoblast invasion
Source: Cell Stem Cell. Author manuscript; Available in PMC 2024 Oct 16. (PMC7616712; doi:10.1016/j.stem.2024.09.004)
Supplement: Supplemental Information [file EMS199345-supplement-Supplemental_Information.pdf]

Supplemental video 1: postTSCs form syncytium by endoreduplication, Related to Figure 3J and Figure S3H,I.

Live IF imaging of marmoset postTSCs tagged with GFP with a nuclear localisation tag (red) and stained with F-actin binding LifeACT (green).

Supplemental Information

Table S1: primer sequences, related to Figure 7J and Figure S2B

Table S2: image quantification data, related to Figure 3,4 and Figure S1,2,4-7
